# Alpha-Lipoic Acid and Saxagliptin Attenuate Ferroptosis-Related Ovarian Injury and Modulate FGF21/NOX4–NRF2/HO-1 Signaling in Letrozole-Induced Polycystic Ovary Syndrome

**DOI:** 10.3390/ijms27219779

**Published:** 2026-10-30

**Authors:** Eman H. Yousef, Mona H. Zohny, Ebtsam S. Abdel-lah, Mostafa E. Metawee, Hader I. Sakr, Mohamed M. Salama

**Affiliations:** 1 Department of Pharmacology and Biochemistry (Biochemistry), Faculty of Pharmacy, Horus University-Egypt, New Damietta 34518, Egypt; ehamdy@horus.edu.eg; 2 Department of Pharmacology and Biochemistry, Faculty of Pharmacy, Delta University for Science and Technology, Gamasa 11152, Egypt; muna.zohny@deltauniv.edu.eg (M.H.Z.); dr.mohamed.salama@hotmail.com (M.M.S.); 3 Department of Pharmacology, Faculty of Veterinary Medicine, Assiut University, Assiut 71526, Egypt; ebtsam_saber@aun.edu.eg; 4 Department of Pharmacology, School of Veterinary Medicine, Badr University in Assiut (BUA), Naser City 11829, Egypt; 5 Department of Histology, Faculty of Medicine (Boys), Al-Azhar University, Cairo 11884, Egypt; histology3.jed@bmc.edu.sa; 6 Department of Histology, General Medicine Practice Program, Batterjee Medical College, Jeddah 21442, Saudi Arabia; 7 Department of Medical Physiology, Faculty of Medicine, Cairo University, Cairo 12613, Egypt; 8 Department of Medical Physiology, General Medicine Practice Program, Batterjee Medical College, Jeddah 21442, Saudi Arabia

**Keywords:** polycystic ovary syndrome, alpha-lipoic acid, saxagliptin, FGF21, NOX4, NRF2, ferroptosis

## Abstract

Polycystic ovary syndrome (PCOS) is a complex endocrine-metabolic disorder characterized by ovarian dysfunction, hormonal imbalance, and emerging ferroptosis-related abnormalities. This study investigated the effects of alpha-lipoic acid (ALA), saxagliptin (Saxa), and their combination in a letrozole-induced PCOS model, with focus on the ovarian FGF21/NOX4–NRF2/HO-1 and ferroptosis-related pathways. PCOS was induced in female rats by oral letrozole administration for 21 days. Rats were allocated into control, PCOS, ALA, Saxa, and ALA/Saxa groups, followed by 14 days of treatment. Body weight, ovarian index, ovarian histopathology, reproductive hormones, oxidative stress markers, ovarian FGF21 and NOX4 expression, NRF2/HO-1 signaling, and ferroptosis-related markers, including Fe^2+^, GPX4, and SLC7A11, were assessed. Letrozole induced weight gain, ovarian hypertrophy, follicular disruption, endocrine imbalance, and ferroptosis-related alterations, marked by increased ROS, MDA, Fe^2+^, and NOX4, alongside reduced FGF21, NRF2/HO-1, GPX4, and SLC7A11. ALA or Saxa attenuated these alterations, whereas ALA/Saxa produced greater improvements in ovarian morphology, hormonal status, redox homeostasis, and ferroptosis-related markers. These effects were associated with FGF21 upregulation, NOX4 suppression, enhanced NRF2/HO-1 signaling, and preservation of GPX4 and SLC7A11. In conclusion, ALA/Saxa therapy markedly ameliorated letrozole-induced PCOS in rats, accompanied by restoration of ovarian FGF21/NOX4 homeostasis, associated with enhancement of NRF2/HO-1 antioxidant responses, and attenuation of ferroptosis-related alterations.

## 1. Introduction

Polycystic ovary syndrome (PCOS) is the most common endocrine-metabolic disorder in reproductive-aged women, affecting approximately 6–13% worldwide. According to the 2003 Rotterdam criteria, PCOS is diagnosed when at least two of the following features are present: hyperandrogenism, oligo- or anovulation, and polycystic ovarian morphology [1,2]. Beyond reproductive dysfunction, PCOS is frequently accompanied by increased adiposity, insulin resistance, impaired glucose and lipid metabolism, oxidative stress, and psychological comorbidities such as depression and anxiety, collectively contributing to its considerable clinical burden [1,3,4,5]. Despite advances in understanding PCOS pathophysiology, the molecular mechanisms underlying its associated ovarian and metabolic disturbances remain incompletely understood. Current management is individualized according to clinical manifestations, metabolic characteristics, and reproductive goals, and includes lifestyle modifications, hormonal therapies, ovulation-inducing agents, and insulin sensitizers [2,6]. Among these, metformin is widely used to improve metabolic and reproductive abnormalities; however, its administration may be limited by gastrointestinal intolerance and potential vitamin B12 deficiency during long-term treatment [2,7,8,9,10]. These limitations, together with the multifactorial nature of PCOS, highlight the need to investigate additional therapeutic approaches targeting the molecular processes associated with ovarian dysfunction.

Oxidative stress has been increasingly recognized as a potential contributor to PCOS-associated ovarian injury [11], raising interest in its relationship with ferroptosis. Ferroptosis is a regulated form of iron-dependent programmed cell death characterized by intracellular iron overload and excessive accumulation of reactive oxygen species (ROS), resulting in lipid peroxidation [12,13]. This process is tightly regulated by antioxidant defense systems, which include glutathione peroxidase 4 (GPX4), GTP cyclohydrolase 1 (GCH1), dihydroorotate dehydrogenase (DHODH), and ferroptosis suppressor protein 1 (FSP1). Among these regulators, GPX4 is a central selenoenzyme that detoxifies phospholipid hydroperoxides and serves as a major regulator of ferroptosis [14]. Although ferroptosis has been extensively investigated in ischemia–reperfusion injury, neurodegeneration, and tumor development [15,16], emerging evidence also implicates ferroptosis-related processes in female reproductive disorders, particularly those involving ovarian granulosa cell injury and impaired reproductive function [17,18,19]. Consistent with increased oxidative stress in PCOS, a meta-analysis demonstrated reduced circulating glutathione (GSH) levels and elevated malondialdehyde (MDA) concentrations in women with PCOS compared with age- and body mass index-matched controls [20]. Furthermore, experimental studies have associated PCOS-related ovarian dysfunction with alterations in iron homeostasis, lipid peroxidation, and ferroptosis-related regulators, including GPX4 and solute carrier family 7 member 11 (SLC7A11) [21,22]. These observations suggest that oxidative stress and ferroptosis-related dysregulation represent potentially important processes in PCOS-associated ovarian injury, although their precise mechanistic contributions require further investigation.

Within this context, fibroblast growth factor 21 (FGF21) and nicotinamide adenine dinucleotide phosphate oxidase 4 (NOX4) have attracted attention because of their reported involvement in stress, mitochondrial dysfunction, and ferroptosis-related ovarian injury. FGF21 is an endocrine metabolic regulator produced predominantly by the liver, with additional expressed in extrahepatic tissues, where it contributes to glucose and lipid metabolism, mitochondrial function, and redox homeostasis [23]. Emerging evidence suggests that FGF21 attenuates ferroptosis by reducing lipid ROS accumulation and engaging NRF2-dependent antioxidant signaling [24,25]. In contrast, NOX4 is a major enzymatic source of intracellular ROS and has been implicated in oxidative stress, granulosa cell injury, and ovarian dysfunction in experimental PCOS model [26]. NOX4-derived ROS further promotes lipid peroxidation and ferroptosis-related cellular damage [27]. Together, current evidence positions FGF21 and NOX4 as complementary metabolic and redox-related regulators linking oxidative stress, NRF2/HO-1 signaling, and ferroptosis-related ovarian injury.

Alpha-lipoic acid (ALA) is a naturally occurring organosulfur compound found in plants and animals and functions as an essential mitochondrial cofactor involved in oxidative metabolism and energy production [28,29]. It has been widely investigated for the management of diabetic complications and exhibits multiple biological activities, including restoration of endogenous antioxidant defenses, chelation of metal ions, reduction in oxidative protein damage, and suppression of inflammatory signaling through inhibition of the nuclear factor-κB (NF-κB) pathway [30,31,32,33,34,35]. In addition, ALA contributes to the regulation of glucose and lipid metabolism [36]. Importantly, previous experimental evidence demonstrated that ALA increases FGF21 expression [37], while FGF21 has been associated with protection against oxidative stress and ferroptosis-related injury [24]. These findings suggest a potential relationship between the biological effects of ALA and FGF21-associated protective responses, providing a rationale for investigating its effects on ovarian FGF21 levels and related redox alterations in PCOS.

Saxagliptin (Saxa) is an orally active anti-diabetic agent that acts as a potent, selective, reversible, and competitive dipeptidyl peptidase-4 (DPP-4) inhibitor [38]. By inhibiting DPP-4, Saxa enhances incretin activity, particularly glucagon-like peptide-1 (GLP-1) and glucose-dependent insulinotropic polypeptide (GIP), thereby improving glucose homeostasis [39]. Beyond glycemic control, Saxa has been reported to enhance insulin sensitivity, improve lipid profiles, and regulate metabolic disturbances [40]. Saxa has also been reported to exert antioxidant and anti-inflammatory effects through modulation of the NRF2/HO-1 and NF-κB pathways in experimental acute lung injury [41], while NRF2/HO-1 signaling is widely implicated in protection against ischemia–reperfusion injury [42]. Moreover, Saxa has been reported to reduce NOX4 under specific experimental conditions [43]. Considering the involvement of NOX4-associated ROS generation in oxidative stress and ovarian injury in PCOS [26], these observations provide a rationale for examining whether Saxa treatment is accompanied by changes in ovarian NOX4 levels and ferroptosis-related parameters.

Although ALA and Saxa have individually demonstrated antioxidant and metabolic effects, their combined effects on PCOS-associated ovarian injury, particularly in relation to FGF21, NOX4, and ferroptosis-related alterations, remain insufficiently investigated. Given their potential to influence distinct but complementary redox-related processes, we hypothesized that their combined administration may provide greater protection against ovarian dysfunction than either agent alone. Therefore, the present study aimed to evaluate the individual and combined effects of ALA and Saxa on ovarian histopathological and hormonal alterations, oxidative stress, and ferroptosis-related parameters in letrozole-induced PCOS rats, with particular emphasis on FGF21, NOX4, and NRF2/HO-1-related antioxidant responses. Exploratory molecular docking was additionally performed to predict potential interactions with selected regulatory proteins associated with these processes.

## 2. Results

### 2.1. ALA/Saxa Attenuated Weight Gain and Ovarian Hypertrophy

**Figure 1:**
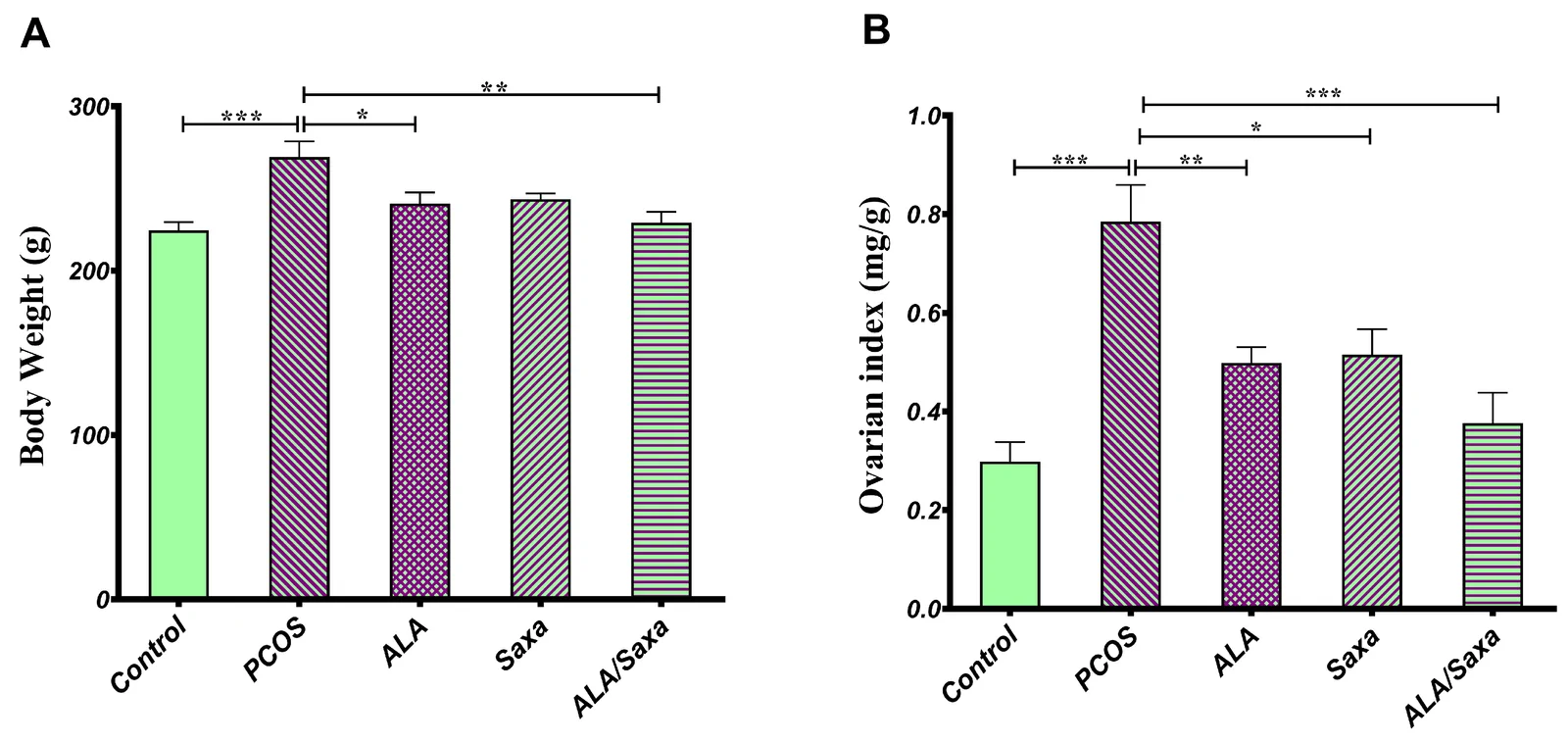
ALA/Saxa ameliorated letrozole-induced PCOS in rats. (**A**) Body weight (g). (**B**) Ovarian index (mg/g). Data are presented as mean ± SEM. Statistical significance was determined by one-way ANOVA followed by Tukey’s multiple-comparisons post hoc test. Brackets indicate the groups being compared. * *p* < 0.05 versus PCOS, ** *p* < 0.01 versus PCOS, and *** *p* < 0.001 versus PCOS.

Letrozole administration at 1 mg/kg/day for 3 weeks markedly increased body weight by 1.2-fold and ovarian index by 2.6-fold compared with the control group (*p* < 0.001), indicating the development of PCOS-like metabolic and ovarian alterations. Compared with untreated PCOS rats, ALA and ALA/Saxa significantly reduced body weight by 1.1-fold and 1.17-fold, respectively (*p* < 0.05 and *p* < 0.01), whereas Saxa alone had no significant effect on body weight. In contrast, ovarian index was significantly decreased by ALA, Saxa, and ALA/Saxa by 1.6-fold, 1.5-fold, and 2.1-fold, respectively, relative to PCOS group (*p* < 0.01, *p* < 0.05, and *p* < 0.001). Notably, the combined ALA/Saxa treatment showed the greatest overall reduction, suggesting marked improvement in PCOS-associated weight gain and ovarian hypertrophy (Figure 1).

### 2.2. ALA/Saxa Ameliorated Ovarian Histopathological Damage and Endocrine Dysfunction

**Figure 2:**
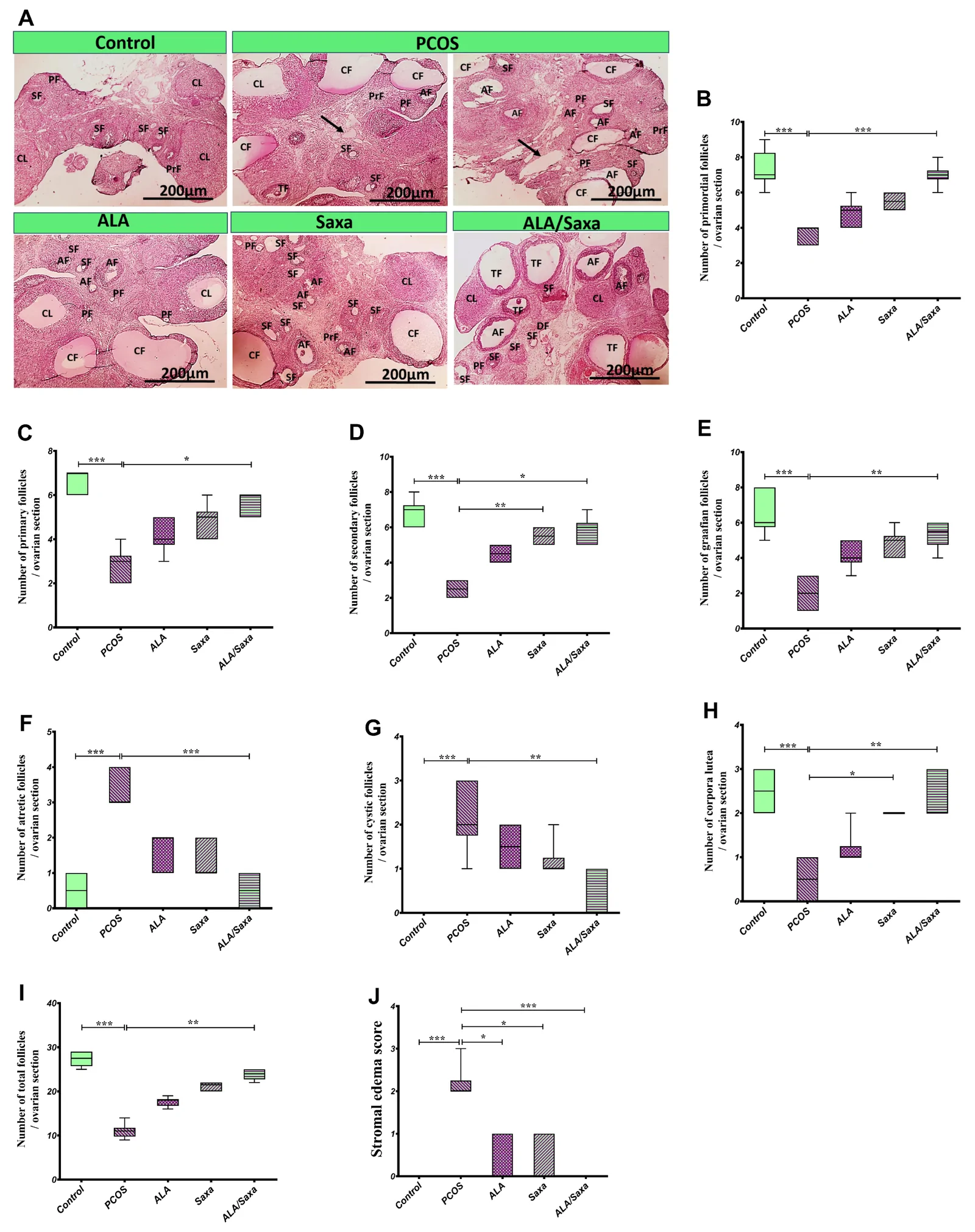
ALA/Saxa ameliorated ovarian histopathological damage. (**A**) Representative H&E-stained ovarian sections from different experimental groups. PrF, primordial follicles; PF, primary follicles; SF, secondary follicles; TF, tertiary follicles; CL, corpora lutea; CF, cystic follicles; AF, atretic follicles; black arrows, stromal edema. Magnification ×40, scale bar = 200 µm. (**B**–**J**) Quantitative evaluation of ovarian histopathological features, including (**B**) primordial follicles, (**C**) primary follicles, (**D**) secondary follicles, (**E**) graafian follicles, (**F**) atretic follicles, (**G**) cystic follicles, (**H**) corpora lutea, (**I**) total follicles, expressed as the mean number per ovarian section. (**J**) Stromal edema, expressed as the mean semi-quantitative score (0–3). Data are presented as median and interquartile range (IQR), with whiskers indicating the minimum and maximum. Statistical significance was determined by the Kruskal–Wallis test followed by Dunn’s multiple-comparisons test. Brackets indicate the groups being compared. * *p* < 0.05 versus PCOS, ** *p* < 0.01 versus PCOS, and *** *p* < 0.001 versus PCOS.

H&E-stained ovarian sections from the control group showed preserved cortex and medulla, with multiple developing follicles, corpora lutea, and no cystic follicles. In contrast, the PCOS group showed ovarian enlargement and marked disruption of follicular organization, manifested by significant reductions in total (*p* < 0.001), primordial (*p* < 0.001), primary (*p* < 0.001), secondary (*p* < 0.001), and mature follicles (*p* < 0.001), together with significant increases in atretic follicles, cystic follicles lined by a monolayer of granulosa cells, and stromal edema (*p* < 0.001) compared with the control group. As monotherapies, ALA and Saxa provided limited overall follicular restoration; however, both reduced stromal edema (*p* < 0.05), and Saxa independently increased secondary follicles and corpora lutea compared with the PCOS group (*p* < 0.05). Notably, the ALA/Saxa combination produced the most consistent histopathological improvement. The combined treatment significantly restored total follicles (*p* < 0.01), specifically elevating primordial (*p* < 0.001), primary (*p* < 0.05), secondary (*p* < 0.05), and mature follicles (*p* < 0.01) alongside increased corpora lutea (*p* < 0.01). Furthermore, the combination markedly suppressed pathological features by reducing atretic follicles (*p* < 0.001), cystic follicles (*p* < 0.01), and stromal edema (*p* < 0.001) compared with the untreated PCOS group (Figure 2).

**Figure 3:**
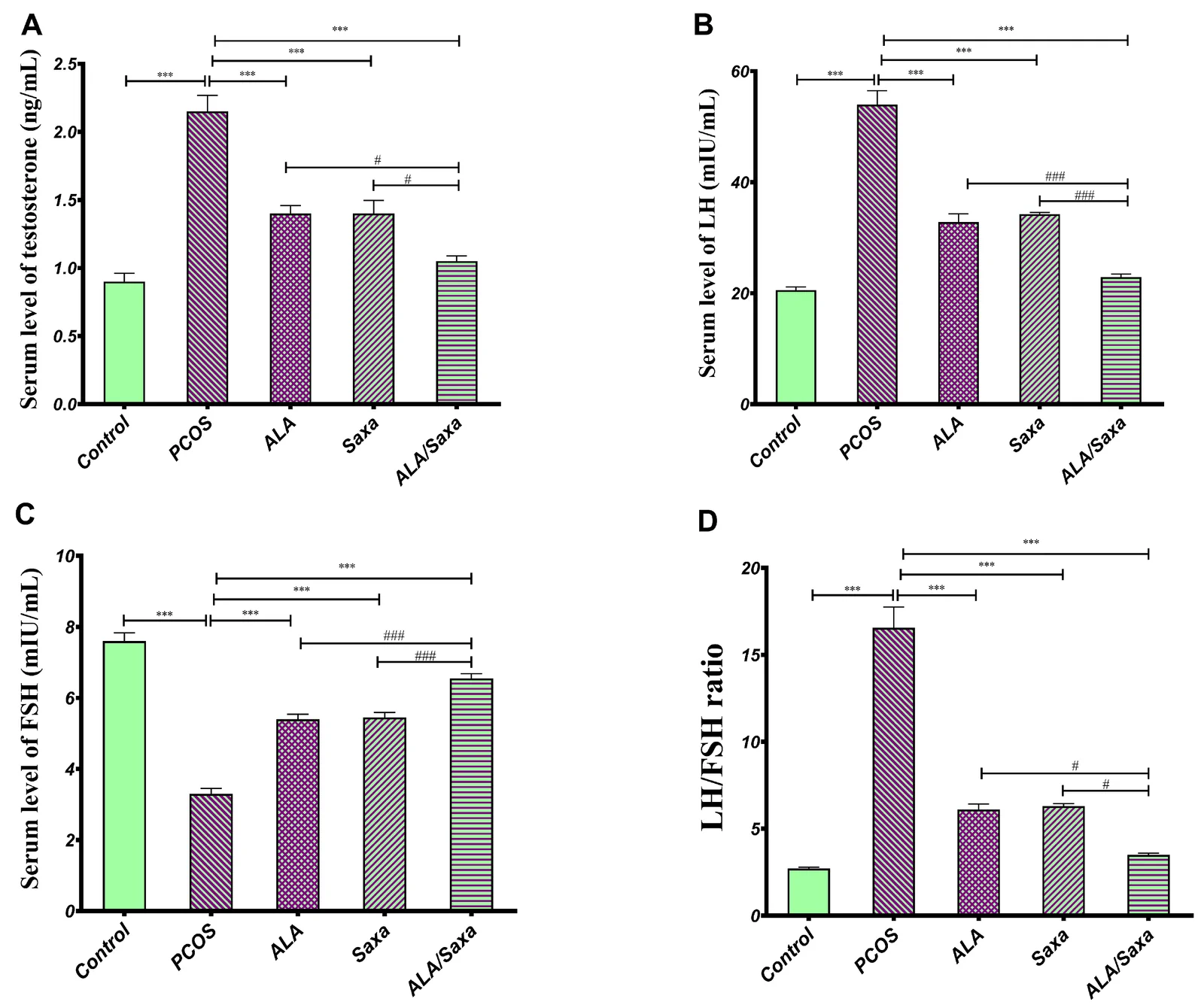
ALA/Saxa alleviated reproductive hormonal imbalance. (**A**) Serum testosterone levels (ng/mL). (**B**) Serum luteinizing hormone (LH) levels (mIU/mL). (**C**) Serum follicle-stimulating hormone (FSH) levels (mIU/mL). (**D**) LH/FSH ratio. Data are presented as mean ± SEM. Statistical significance was determined by one-way ANOVA followed by Tukey’s multiple-comparisons post hoc test. Brackets indicate the groups being compared. *** *p* < 0.001 versus PCOS, # *p* < 0.05 versus ALA/Saxa, and ### *p* < 0.001 versus ALA/Saxa.

Letrozole-induced PCOS significantly increased serum testosterone, LH, and the LH/FSH ratio by 2.4-fold, 2.6-fold, and 6.1-fold, respectively, while decreasing FSH levels by 2.3-fold compared with the control group (*p* < 0.001), indicating reproductive hormonal imbalance. Compared with the PCOS group, treatment with ALA, Saxa, and ALA/Saxa significantly reduced testosterone levels by 1.5-fold, 1.5-fold, and 2.04-fold, respectively (*p* < 0.001). The ALA/Saxa combination further reduced testosterone levels by 1.33-fold compared with either ALA or Saxa alone (*p* < 0.05). Similarly, ALA, Saxa, and ALA/Saxa significantly reduced LH levels by 1.64-fold, 1.58-fold, and 2.36-fold, respectively, compared with the PCOS group (*p* < 0.001), with the combination showing additional reductions of 1.43-fold and 1.49-fold compared with ALA and Saxa, respectively (*p* < 0.001). FSH levels were significantly increased by ALA, Saxa, and ALA/Saxa by 1.64-fold, 1.65-fold, and 1.98-fold, respectively, compared with the PCOS group (*p* < 0.001), and the combination further increased FSH by 1.21-fold and 1.2-fold compared with ALA and Saxa, respectively (*p* < 0.001). The LH/FSH ratio was also significantly reduced by ALA, Saxa, and ALA/Saxa by 2.7-fold, 2.63-fold, and 4.73-fold, respectively, compared with the PCOS group (*p* < 0.001), with additional reductions of 1.74-fold and 1.8-fold compared with ALA and Saxa, respectively (*p* < 0.05). These findings indicate that ALA/Saxa alleviated reproductive hormonal imbalance, with the combination producing greater improvement than either monotherapy (Figure 3).

### 2.3. Molecular Docking Predicts Potential Interactions of ALA and Saxa with Selected Regulatory Proteins

Molecular docking studies were performed to evaluate the predicted interactions of ALA and Saxa with IKKβ (3RZF), SIRT1 (4ZZJ), PPAR-α (6KXX), AMPK (4ZHX), and KEAP1 (4CXT). The predicted binding poses were compared with those of the respective co-crystallized ligands, with detailed ligand–residue interactions and binding affinities presented in Table 1 and Table 2.

**Figure 4:**
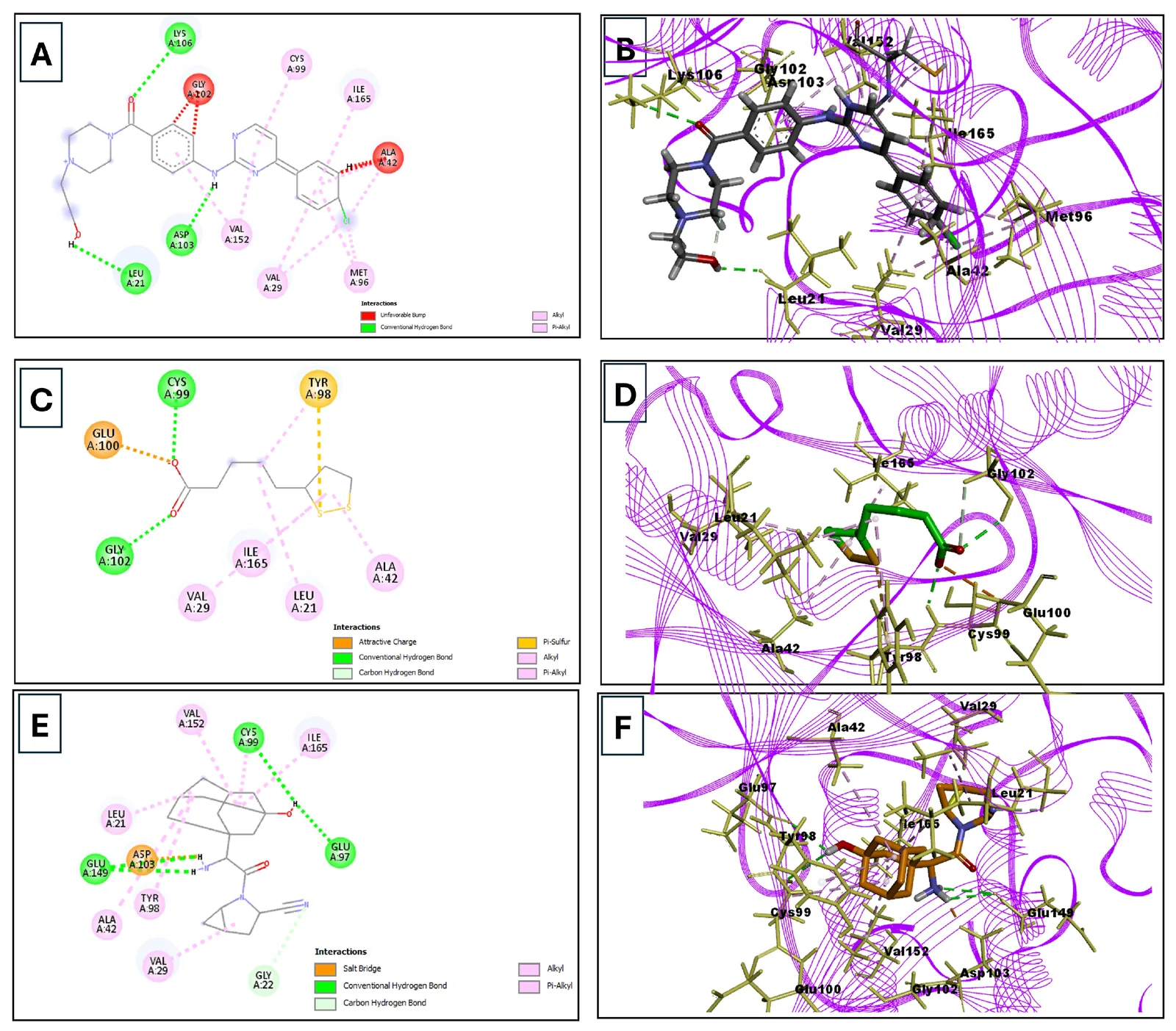
Molecular docking of ALA and Saxa within the XNM-binding pocket of IKKβ (PDB: 3RZF). (**A**) Two-dimensional interaction map of the co-crystallized ligand XNM. (**B**) Three-dimensional binding pose of XNM (gray) within the binding pocket. (**C**) Two-dimensional interaction map of ALA. (**D**) Three-dimensional binding pose of ALA (green) within the XNM-binding pocket. (**E**) Two-dimensional interaction map of Saxa. (**F**) Three-dimensional binding pose of Saxa (orange) within the XNM-binding pocket.

For IKKβ (PDB: 3RZF), the co-crystallized ligand XNM occupied the ATP-binding pocket and interacted with Leu21, Asp103, and Lys106 through hydrogen bonding, together with hydrophobic contacts involving Val29, Ala42, Met96, Cys99, Val152, and Ile165 (Figure 4A,B; Table 1). ALA was predicted to occupy the same binding pocket, forming hydrogen bonds with Cys99 and Gly102 and sharing hydrophobic contacts with the XNM-binding region, including Leu21, Val29, Ala42, and Ile165. Saxa was also predicted to occupy the XNM-binding pocket, with interactions involving Cys99 and several residues shared with the co-crystallized ligand, including Leu21, Val29, Ala42, Val152, and Ile165. The predicted binding affinities of ALA and Saxa were −5.1 and −7.4 kcal/mol, respectively (Figure 4C–F; Table 2).

**Figure 5:**
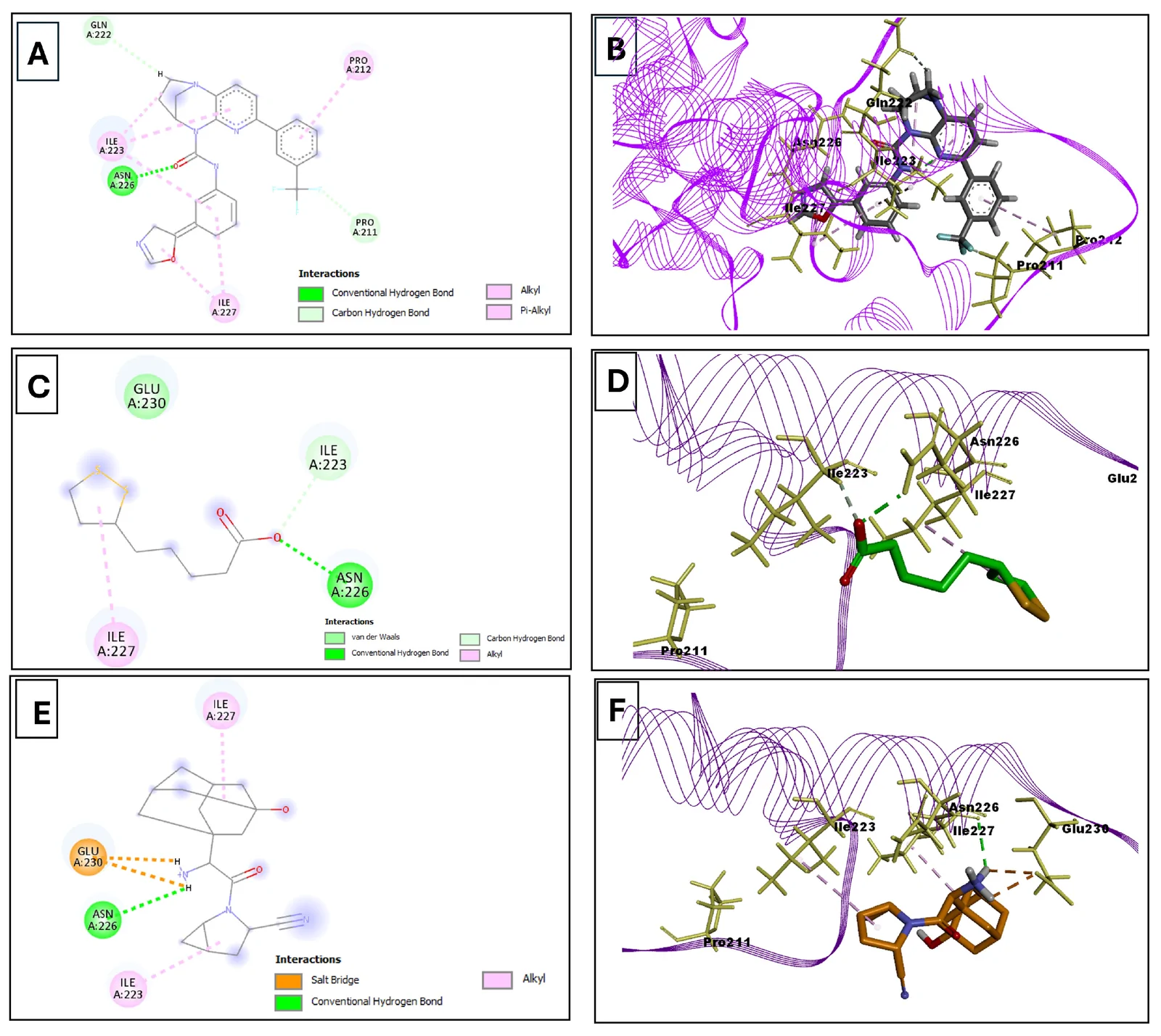
Molecular docking of ALA and Saxa within the 4TQ-binding pocket of SIRT1 (PDB: 4ZZJ). (**A**) Two-dimensional interaction map of the co-crystallized ligand 4TQ. (**B**) Three-dimensional binding pose of 4TQ (gray) within the binding pocket. (**C**) Two-dimensional representations of the predicted ALA binding pose within the binding pocket. (**D**) Three-dimensional representations of the predicted ALA (green) binding pose within the binding pocket. (**E**) Two-dimensional representations of the predicted Saxa binding pose within the binding pocket. (**F**) Three-dimensional representations of the predicted Saxa (orange) binding pose within the binding pocket.

For SIRT1 (PDB: 4ZZJ), the co-crystallized ligand 4TQ occupied the allosteric activator-binding pocket, with a key hydrogen-bond interaction involving Asn226 and hydrophobic contacts with Pro212, Ile223, and Ile227 (Figure 5A,B; Table 1). ALA and Saxa were predicted to occupy the same 4TQ-binding pocket, with both compounds reproducing the interaction with Asn226. ALA additionally interacted with Ile227, whereas Saxa showed hydrophobic interactions with Ile223 and Ile227. The predicted binding affinities were −3.3 kcal/mol for ALA and −4.7 kcal/mol for Saxa (Figure 5C–F; Table 2).

**Figure 6:**
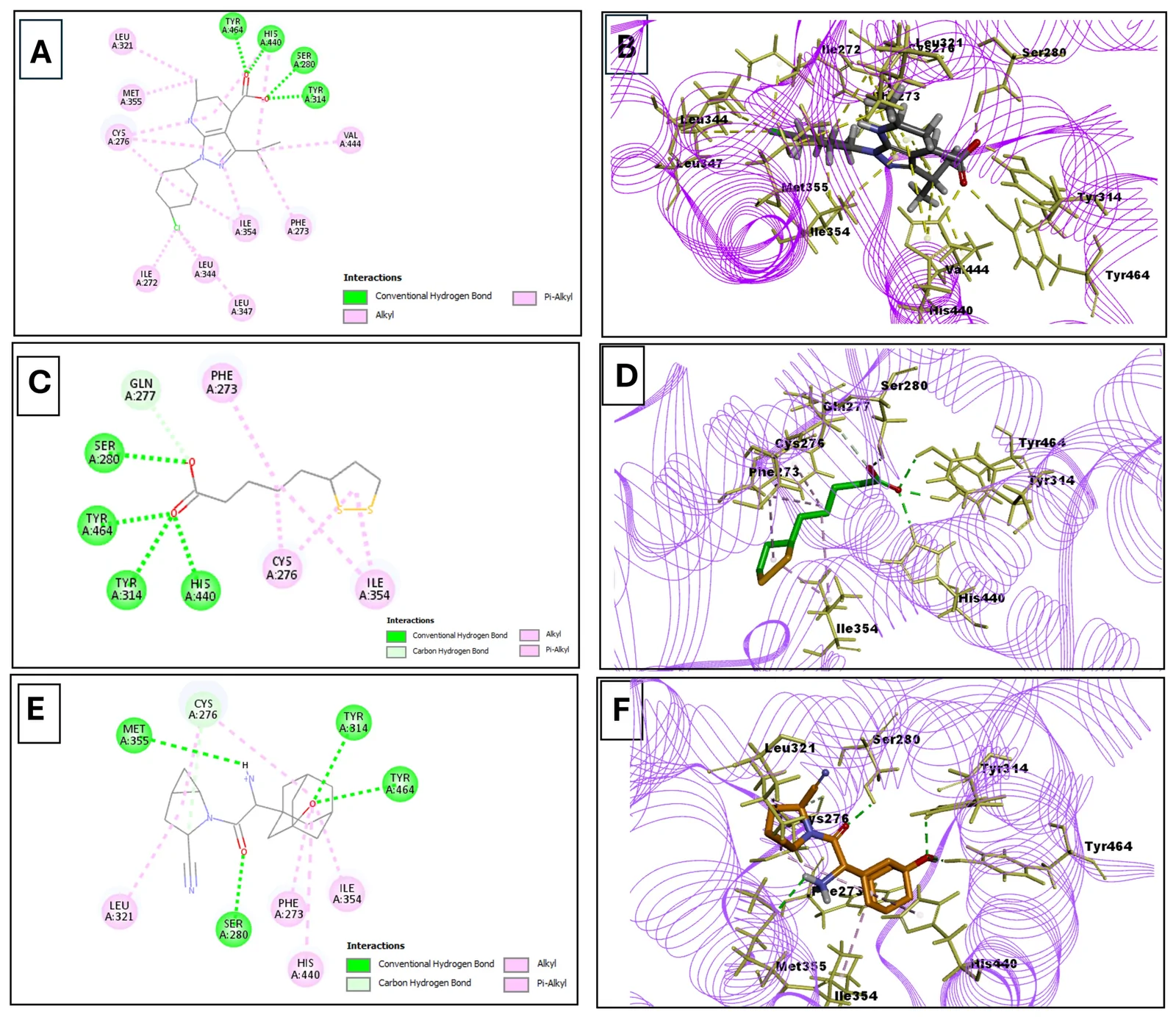
Molecular docking of ALA and Saxa within the T02-binding pocket of PPARα (PDB: 6KXX). (**A**) Two-dimensional interaction map of the co-crystallized ligand T02. (**B**) Three-dimensional binding pose of T02 (gray) within the binding pocket. (**C**) Two-dimensional interaction map of ALA. (**D**) Three-dimensional binding pose of ALA (green) within the T02-binding pocket. (**E**) Two-dimensional interaction map of Saxa. (**F**) Three-dimensional binding pose of Saxa (orange) within the T02-binding pocket.

For PPARα (PDB: 6KXX), the co-crystallized ligand T02 occupied the ligand-binding pocket and formed key hydrogen-bond interactions with Ser280, Tyr314, His440, and Tyr464, together with hydrophobic contacts within the binding cavity (Figure 6A,B; Table 1). ALA was predicted to occupy the same binding pocket and reproduced interactions with all four key residues, Ser280, Tyr314, His440, and Tyr464, in addition to hydrophobic contacts with Cys276, Phe273, and Ile354 (Figure 6C,D; Table 2). Saxa was also predicted to occupy the T02-binding pocket and reproduced interactions with Ser280, Tyr314, and Tyr464, together with additional interactions involving Met355 and hydrophobic contacts within the binding region. The predicted binding affinities were −5.5 kcal/mol for ALA and −4.9 kcal/mol for Saxa (Figure 6E,F; Table 2).

**Figure 7:**
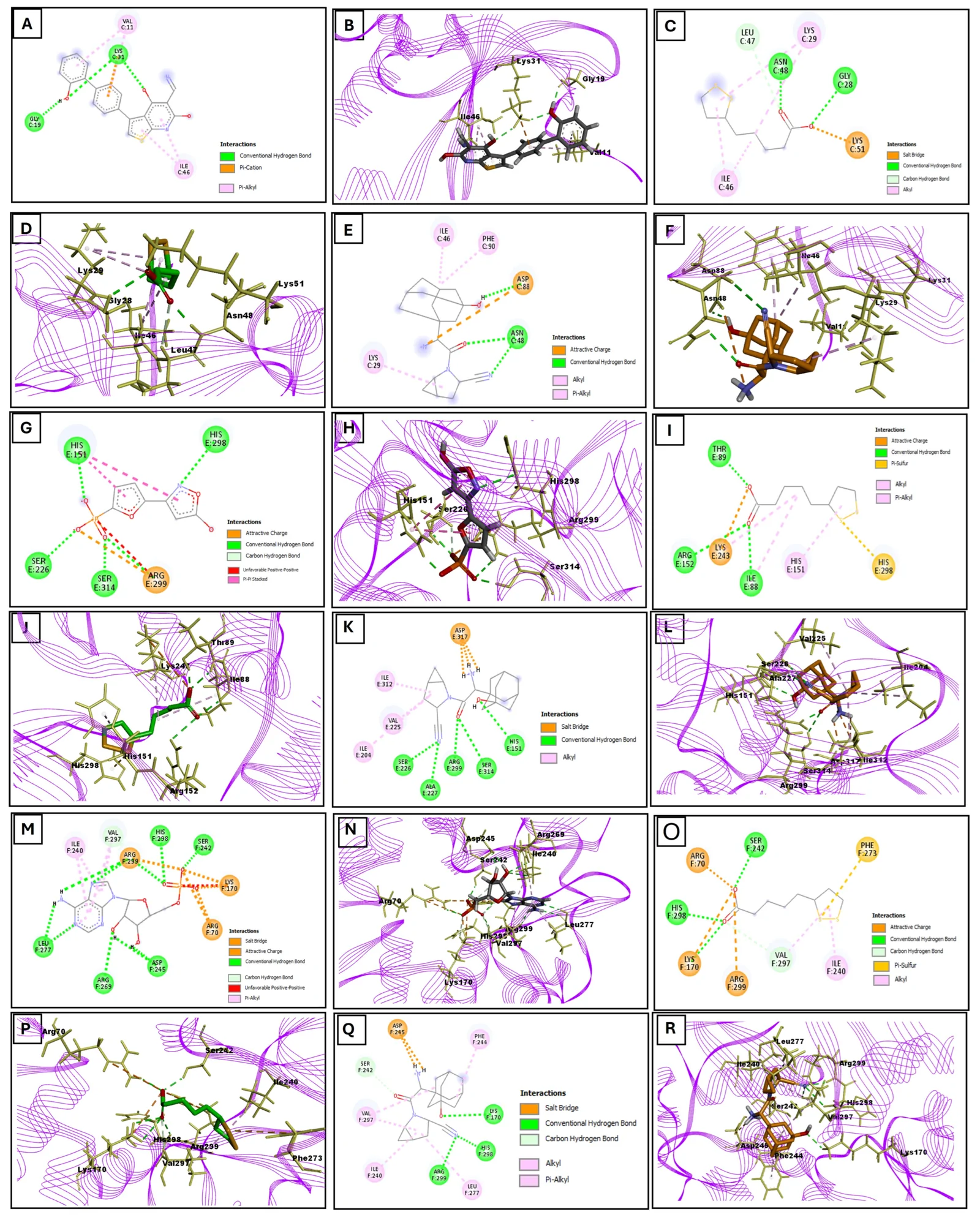
Molecular docking of ALA and Saxa at three binding regions of AMPK (PDB: 4ZHX) corresponding to the co-crystallized ligands C1V, C2Z, and AMP. (**A**) Two-dimensional interaction map of the co-crystallized ligand (C1V) within the AMPK chain C binding pocket. (**B**) Three-dimensional binding pose of C1V (gray) within the AMPK chain C binding pocket. (**C**) Two-dimensional interaction map of ALA within the AMPK chain C binding pocket. (**D**) Three-dimensional binding pose of ALA (green) within the AMPK chain C binding pocket. (**E**) Two-dimensional interaction map of Saxa within the AMPK chain C binding pocket. (**F**) Three-dimensional binding pose of Saxa (orange) within the AMPK chain C binding pocket. (**G**) Two-dimensional interaction map of the co-crystallized ligand (C2Z) within the AMPK chain E binding pocket. (**H**) Three-dimensional binding pose of C2Z (gray) within the AMPK chain E binding pocket. (**I**) Two-dimensional interaction map of ALA within the AMPK chain E binding pocket. (**J**) Three-dimensional binding pose of ALA (green) within the AMPK chain E binding pocket. (**K**) Two-dimensional interaction map of Saxa within the AMPK chain E binding pocket. (**L**) Three-dimensional binding pose of Saxa (orange) within the AMPK chain E binding pocket. (**M**) Two-dimensional interaction map of the co-crystallized ligand (AMP) within the AMPK chain F binding pocket. (**N**) Three-dimensional binding pose of AMP (gray) within the AMPK chain F binding pocket. (**O**) Two-dimensional interaction map of ALA within the AMPK chain F binding pocket. (**P**) Three-dimensional binding pose of ALA (green) within the AMPK chain F binding pocket. (**Q**) Two-dimensional interaction map of Saxa within the AMPK chain F binding pocket. (**R**) Three-dimensional binding pose of Saxa (orange) within the AMPK chain F binding pocket.

For AMPK (PDB: 4ZHX), docking was evaluated at three binding regions corresponding to the co-crystallized ligands C1V, C2Z, and AMP (Figure 7; Table 1 and Table 2). Within the C1V-binding pocket, ALA and Saxa were predicted to interact with residues including Asn48 and Ile46, although their interaction patterns differed from that of C1V. At the C2Z-binding region, ALA interacted with His151 and residues within an adjacent region of the pocket, whereas Saxa reproduced several interactions observed for C2Z, including those involving His151, Ser226, Arg299, and Ser314. Within the AMP-binding region, ALA showed predicted interactions with Arg70, Lys170, Ser242, and His298, whereas Saxa interacted with Lys170, His298, and Arg299 and showed additional contacts within the same binding region. Across the three evaluated AMPK sites, the predicted binding affinities ranged from −3.7 to −5.5 kcal/mol for ALA and from −5.2 to −7.5 kcal/mol for Saxa (Figure 7; Table 2).

**Figure 8:**
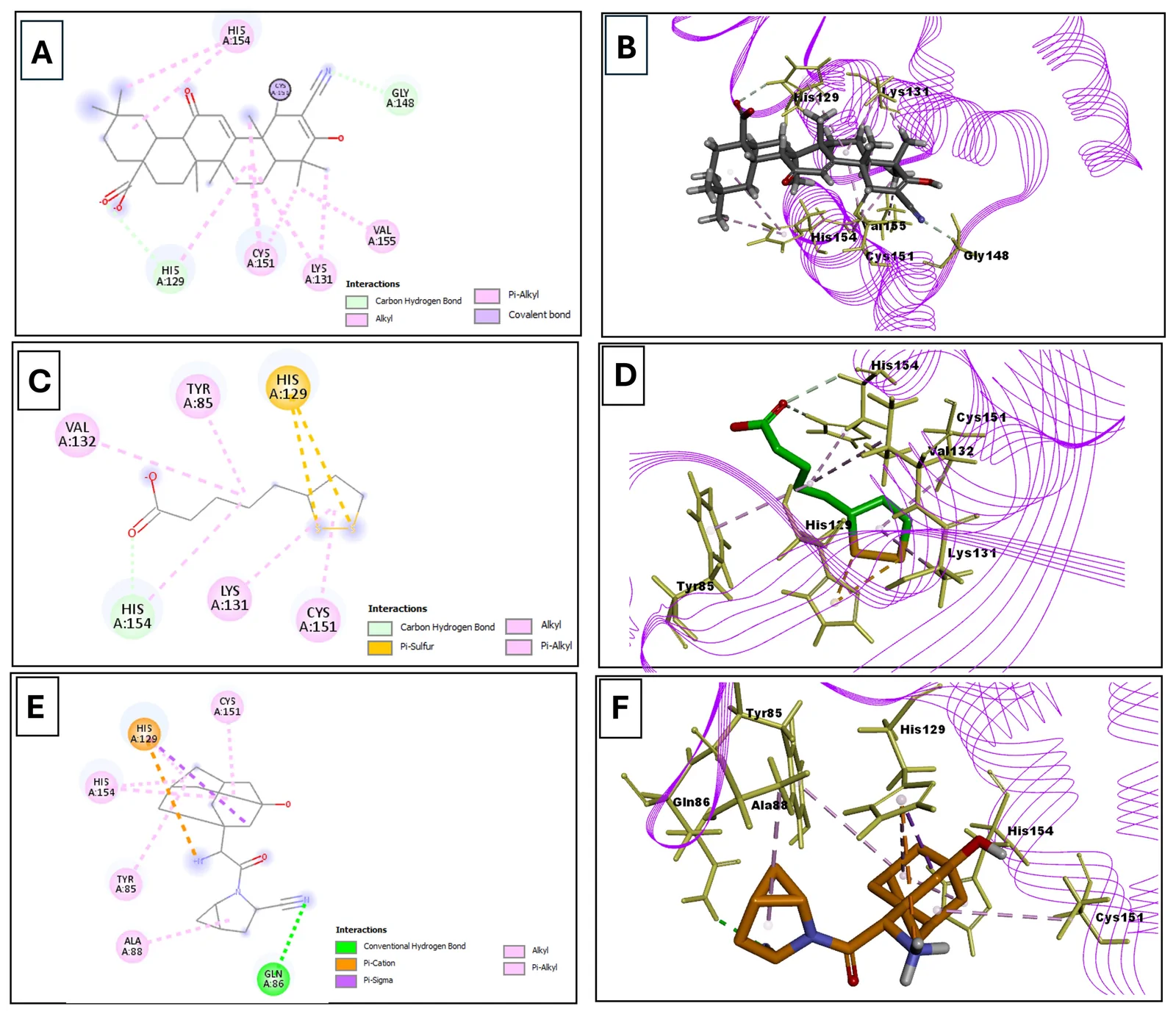
Molecular docking of ALA and Saxa within the SXJ-binding region of the KEAP1 BTB domain (PDB: 4CXT). (**A**) Two-dimensional interaction map of the co-crystallized ligand SXJ. (**B**) Three-dimensional binding pose of SXJ (gray) within the binding region. (**C**) Two-dimensional interaction map of ALA. (**D**) Three-dimensional predicted binding pose of ALA (green) within the SXJ-binding pocket. (**E**) Two-dimensional interaction map of Saxa. (**F**) Three-dimensional binding pose of Saxa (orange) within the SXJ-binding pocket.

For KEAP1 (PDB: 4CXT), the co-crystallized ligand SXJ occupied a binding site within the BTB domain, with hydrophobic interactions involving Lys131, Cys151, Val155, His129, and His154 (Figure 8A,B; Table 1). ALA was predicted to occupy the same binding region and showed hydrophobic interactions with Lys131, Val132, Cys151, Tyr85, and His154. Saxa was also predicted to occupy the SXJ-binding region, forming a hydrogen bond with Gln86 together with hydrophobic interactions involving Tyr85, Ala88, Cys151, His129, and His154. The predicted binding affinities were −4.2 kcal/mol for ALA and −5.2 kcal/mol for Saxa (Figure 8C–F; Table 2).

### 2.4. ALA/Saxa Improved the Dysregulated Ovarian Expression of FGF21 and NOX4

**Figure 9:**
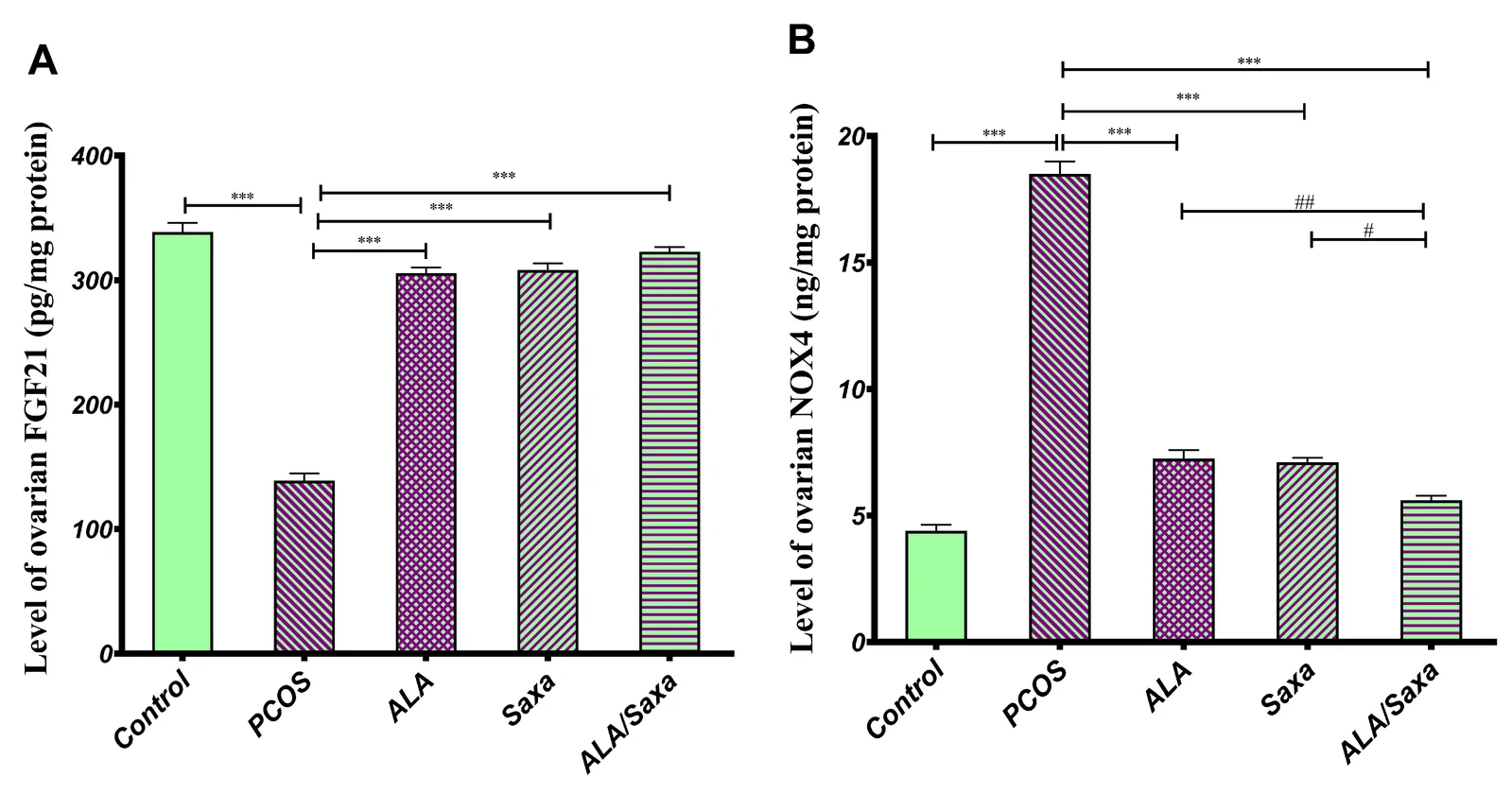
ALA/Saxa modulated ovarian FGF21 and NOX4 expression. (**A**) Ovarian FGF21 levels (pg/mg protein). (**B**) Ovarian NOX4 levels (ng/mg protein). Data are presented as mean ± SEM. Statistical significance was determined by one-way ANOVA followed by Tukey’s multiple-comparisons post hoc test. Brackets indicate the groups being compared. # *p* < 0.05 versus ALA/Saxa, ## *p* < 0.01 versus ALA/Saxa, and *** *p* < 0.001 versus PCOS.

Previous studies have suggested that PCOS-related pathological alterations are associated with dysregulated FGF21 and NOX4 expression [26,44]. Consistent with this, letrozole-induced PCOS significantly decreased ovarian FGF21 levels by 2.44-fold (*p* < 0.001) and increased NOX4 levels by 4.2-fold (*p* < 0.001) compared with the control group. Compared with the PCOS group, treatment with ALA, Saxa, and ALA/Saxa significantly increased FGF21 levels by 2.2-fold, 2.22-fold, and 2.33-fold, respectively (*p* < 0.001). In parallel, ALA, Saxa, and ALA/Saxa significantly reduced NOX4 levels by 2.55-fold, 2.6-fold, and 3.3-fold, respectively, compared with the PCOS group (*p* < 0.001). Moreover, ALA/Saxa further reduced NOX4 levels by 1.3-fold compared with ALA (*p* < 0.01) and by 1.27-fold compared with Saxa (*p* < 0.05), indicating a greater effect of the combined treatment on NOX4 suppression (Figure 9).

### 2.5. ALA/Saxa Enhanced NRF2/HO-1 Signaling and Suppressed Ovarian Redox Injury

**Figure 10:**
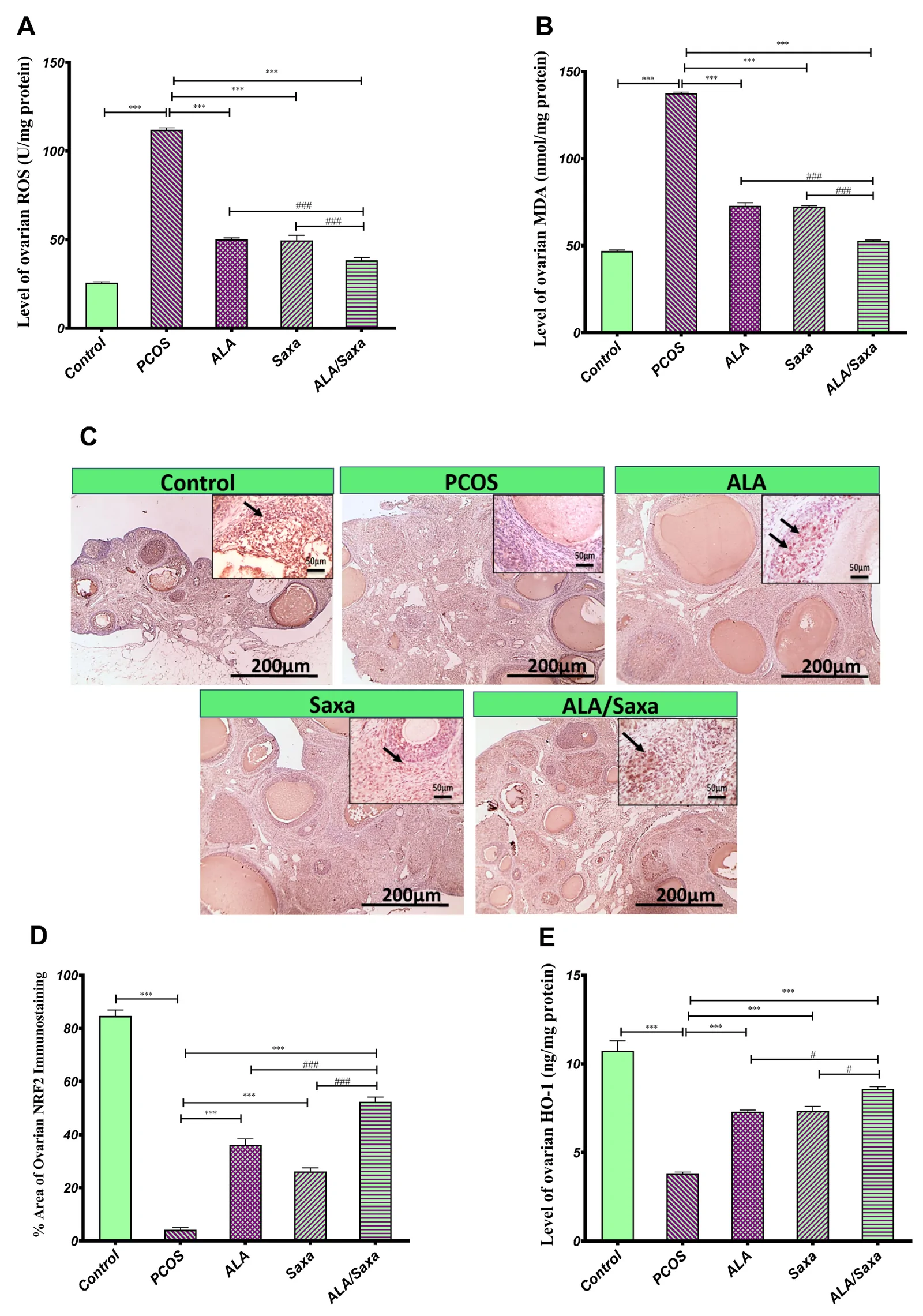
ALA/Saxa alleviated ovarian oxidative stress and enhanced NRF2/HO-1 signaling. (**A**) Ovarian ROS levels (U/mg protein). (**B**) Ovarian MDA levels (nmol/mg protein). (**C**) Representative images of anti-NRF2-stained ovarian sections. Arrows indicate positive brown staining. Sections were counterstained with Mayer’s hematoxylin. Magnification: ×40, scale bar = 200 µm; ×400, scale bar = 50 µm. (**D**) Quantitative analysis of NRF2 immunoreactivity, expressed as percentage-stained area. (**E**) Ovarian HO-1 levels (ng/mg protein). Data are presented as mean ± SEM. Statistical significance was determined by one-way ANOVA followed by Tukey’s multiple-comparisons post hoc test. Brackets indicate the groups being compared. *** *p* < 0.001 versus PCOS, # *p* < 0.05 versus ALA/Saxa, and ### *p* < 0.001 versus ALA/Saxa.

Oxidative stress is closely implicated in PCOS pathogenesis, and NOX4-derived ROS can promote lipid and protein oxidation and activate apoptosis pathway, thereby aggravating ovarian injury [26]. Therefore, ovarian ROS and MDA levels were assessed (Figure 10A,B). Compared with the control group, letrozole-induced PCOS significantly increased ROS and MDA levels by 4.36-fold and 2.93-fold, respectively (*p* < 0.001). Compared with the PCOS group, ALA, Saxa, and ALA/Saxa significantly reduced ROS levels by 2.2-fold, 2.26-fold, and 2.9-fold, respectively (*p* < 0.001). The ALA/Saxa combination further reduced ROS levels by 1.31-fold and 1.29-fold compared with ALA and Saxa, respectively (*p* < 0.001). Similarly, ALA, Saxa, and ALA/Saxa significantly reduced MDA levels by 1.89-fold, 1.9-fold, and 2.6-fold, respectively, compared with the PCOS group (*p* < 0.001). The combination further decreased MDA levels by 1.38-fold and 1.37-fold compared with ALA and Saxa, respectively (*p* < 0.001).

Considering the central role of NRF2/HO-1 signaling in antioxidant defense, spatial NRF2 expression was evaluated by immunohistochemistry in ovarian sections (Figure 10C,D), while total HO-1 levels were quantified by ELISA in ovarian homogenates (Figure 10E). Qualitative assessment revealed that NRF2 immunoreactivity was predominantly localized to follicular, peri-follicular and stromal cells. Compared with the control group, letrozole-induced PCOS markedly reduced NRF2 immunoreactivity within these compartments by 20.3-fold (*p* < 0.001). Compared with the PCOS group, treatment with ALA, Saxa, and ALA/Saxa increased NRF2 immunoreactivity by 8.7-fold, 6.3-fold, and 12.6-fold, respectively (*p* < 0.001), with the combination further increasing it by 1.45-fold and 2.0-fold compared with ALA and Saxa alone, respectively (*p* < 0.001). In parallel, whole-ovarian HO-1 levels were reduced by 2.8-fold in the PCOS group compared with controls (*p* < 0.001) and significantly increased by 1.9-fold, 1.93-fold, and 2.26-fold following treatment with ALA, Saxa, and ALA/Saxa, respectively (*p* < 0.001). The combination therapy further increased HO-1 levels by 1.18-fold and 1.17-fold compared with ALA and Saxa, respectively (*p* < 0.05).

### 2.6. ALA/Saxa Attenuated Ovarian Ferroptosis-Related Alterations in PCOS Rats

**Figure 11:**
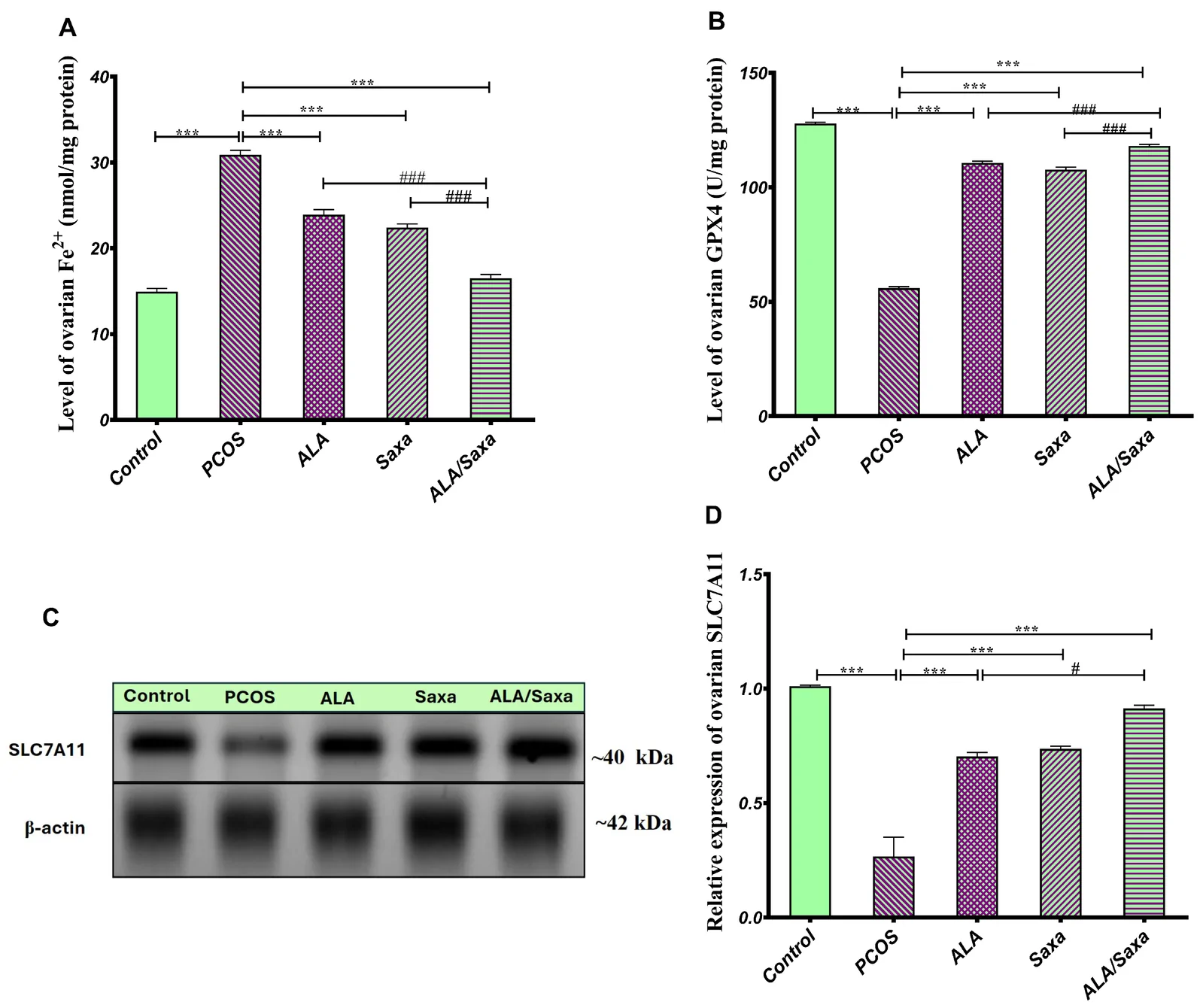
ALA/Saxa attenuated ovarian ferroptosis-related alterations. (**A**) Ovarian Fe^2+^ levels (nmol/mg protein). (**B**) Ovarian GPX4 level (U/mg protein). (**C**) Representative Western blot showing SLC7A11 protein expression. (**D**) Quantitative densitometric analysis of SLC7A11 expression. Data are presented as mean ± SEM. Statistical significance was determined by one-way ANOVA followed by Tukey’s multiple-comparisons post hoc test. Brackets indicate the groups being compared. *** *p* < 0.001 versus PCOS, # *p* < 0.05 versus ALA/Saxa, and ### *p* < 0.001 versus ALA/Saxa.

PCOS-related ovarian dysfunction has been associated with increased programmed cell death, including ferroptosis [45]. Previous studies have also suggested that some PCOS therapies, such as metformin, may exert beneficial effects through ferroptosis inhibition [46]. Therefore, ovarian Fe^2+^, GPX4, and SLC7A11 levels were assessed (Figure 11). Compared with the control group, letrozole-induced PCOS significantly increased Fe^2+^ levels by 2.1-fold (*p* < 0.001), while reducing GPX4 level and SLC7A11 expression by 2.29-fold and 3.79-fold, respectively (*p* < 0.001). Compared with the PCOS group, ALA, Saxa, and ALA/Saxa significantly reduced Fe^2+^ levels by 1.29-fold, 1.38-fold, and 1.87-fold, respectively (*p* < 0.001). The ALA/Saxa combination further reduced Fe^2+^ levels by 1.45-fold and 1.36-fold compared with ALA and Saxa, respectively (*p* < 0.001). In parallel, ALA, Saxa, and ALA/Saxa significantly increased GPX4 level by 1.98-fold, 1.93-fold, and 2.11-fold, respectively, compared with the PCOS group (*p* < 0.001), with the combination further increasing GPX4 by 1.07-fold and 1.1-fold compared with ALA and Saxa, respectively (*p* < 0.001). SLC7A11 expression was also significantly increased by ALA, Saxa, and ALA/Saxa by 2.64-fold, 2.76-fold, and 3.4-fold, respectively, compared with the PCOS group (*p* < 0.001). The ALA/Saxa combination further increased SLC7A11 expression by 1.3-fold compared with ALA alone (*p* < 0.05).

### 2.7. Multivariable Data Analyses

**Figure 12:**
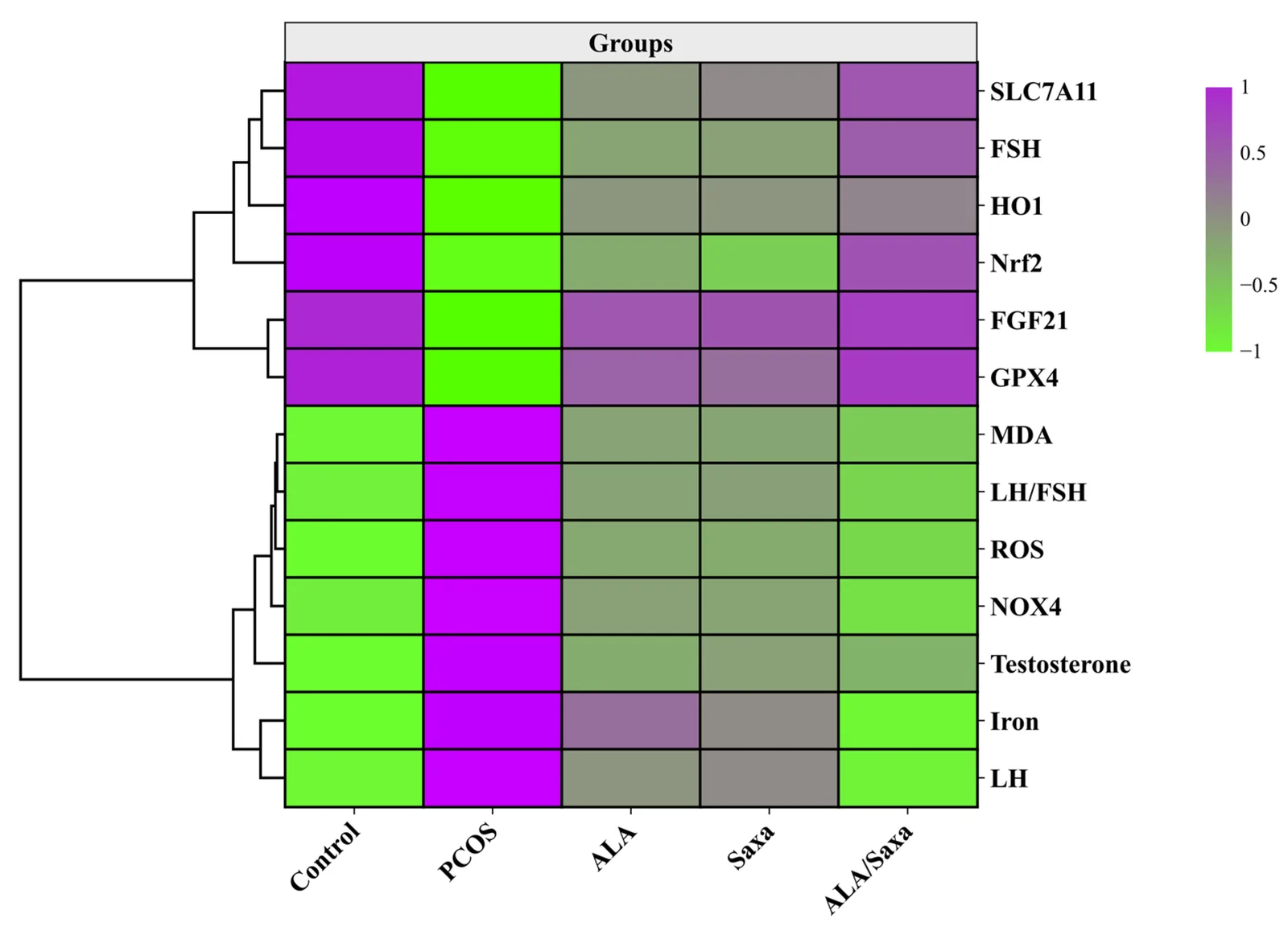
Heatmap representing hierarchical clustering of hormonal, oxidative stress, ferroptosis-related, and molecular markers across the control, PCOS, ALA-treated, Saxa-treated, and ALA/Saxa-treated groups. Color gradients indicate relative expression levels (purple = upregulation, green = downregulation; Z-score normalized). The PCOS group exhibited elevated levels of oxidative stress, ferroptosis, and endocrine disturbance markers, including MDA, ROS, NOX4, testosterone, LH, LH/FSH ratio, and iron. In contrast, these markers were markedly reduced in the treated groups, particularly in the ALA/Saxa combination group. Conversely, FSH, FGF21, NRF2, HO-1, GPX4, and SLC7A11 were downregulated in the PCOS group and progressively restored following treatment with ALA or Saxa, with the combination therapy showing the most pronounced upregulation. The clustering pattern demonstrates the ability of ALA and Saxa, especially in combination, to ameliorate PCOS-associated oxidative stress, ferroptosis, and hormonal imbalance while restoring antioxidant defense mechanisms.

The heatmap provided an integrated visualization of the relative patterns of change across the measured hormonal, oxidative stress, ferroptosis-related, and molecular parameters (Figure 12). The PCOS group displayed a pattern distinct from the control group, whereas ALA, Saxa, and their combination shifted the overall profiles toward the control pattern. The combined treatment showed broader normalization across the measured parameters.

## 3. Materials and Methods

### 3.1. In Silico Molecular Docking

Molecular docking analysis was performed to evaluate the binding interactions of ALA and Saxa with AMP-activated protein kinase (AMPK), Peroxisome Proliferator-Activated Receptor alpha (PPARα), Sirtuin 1 (SIRT1), Inhibitory kappa B kinase beta (IKKβ), and Kelch-like ECH-associated protein 1 (KEAP1) using AutoDock Vina (version 1.1.2) and AutoDock Tools, following previously established protocols [47]. The docking targets were selected based on their established regulatory relationships with the molecular pathways experimentally investigated in the present study. Although FGF21, NOX4, and NRF2 were directly evaluated in ovarian tissues, suitable experimentally resolved structures containing appropriate co-crystallized small-molecule ligands for the conventional structure-based docking approach employed in this study were not identified for these targets. Therefore, structurally suitable regulatory proteins with established biological links to these pathways were selected. AMPK, SIRT1, and PPARα have been implicated in the regulation of FGF21 expression [48,49,50,51,52], whereas IKKβ was selected based on the involvement of NF-κB signaling in NOX4 transcriptional regulation [53,54]. KEAP1 was included as the principal negative regulator of NRF2 [55,56]. Accordingly, these proteins were used as structurally suitable targets to explore potential interactions with ALA and Saxa. The crystal structure of AMPK (PDB ID: 4ZHX), PPARα (PDB ID: 6KXX), SIRT1 (PDB ID: 4ZZJ), IKKβ (PDB ID: 3RZF) and KEAP1 (PDB ID: 4CXT) were retrieved from the Protein Data Bank. Prior to docking, the protein structure was prepared by removing water molecules and non-essential co-crystallized ligands, followed by the addition of hydrogen atoms to ensure appropriate ionization and protonation states.

The three-dimensional structure of the ALA and Saxa were obtained from the PubChem database and subjected to structural optimization, including bond order correction, charge assignment, hydrogen addition, and energy minimization. Both protein and ligand structures were converted to PDBQT format for compatibility with AutoDock. Docking simulations were performed at the active site defined by the co-crystallized ligand, using a grid box with an appropriate search radius (Table 3). The resulting binding poses and intermolecular interactions were analyzed and visualized using Discovery Studio Visualizer (version 21.1.0.20298).

### 3.2. Drugs and Chemicals

Letrozole (Femara^®^; 2.5 mg/tablet) was procured from Novartis Pharmaceuticals (Basel, Switzerland). ALA was obtained from Sigma-Aldrich (St. Louis, MO, USA), and Saxa (Onglyza^®^; Saxa; 5 mg/tablet) was supplied by Mash Premiere Pharmaceutical Company (Cairo, Egypt). For pharmacological induction and treatment, letrozole and Saxa were freshly suspended in 0.5% (*w*/*v*) carboxymethyl cellulose (CMC) immediately prior to oral administration. All other reagents were of analytical grade and used as received.

### 3.3. Animals

Thirty adult female Rattus norvegicus rats (200 ± 20 g) were obtained from the Egyptian Organization for Biological Products and Vaccines (Giza, Egypt) and housed in polypropylene cages under standardized environmental conditions, including a 12 h light/dark cycle and a temperature of 25 ± 3 °C, with unrestricted access to food and water. Animals were allowed to acclimatize for 7 days prior to the start of the experiment. All procedures were reviewed and approved by the Research Ethics Committee of Horus University, Egypt (Ethical code: PH-2026-018) and were conducted in strict accordance with the National Institutes of Health (NIH) Guide for the Care and Use of Laboratory Animals (NIH Publication No. 85–23, revised 2011) and the ARRIVE guidelines to ensure ethical compliance and reproducibility. An a priori power analysis was performed using G*Power software (version 3.1.9.4; Heinrich Heine University Düsseldorf, Düsseldorf, Germany) to determine the required sample size for a one-way ANOVA across the five experimental groups. The calculation was based on an effect size of f = 0.70, an α error probability of 0.05, and a statistical power (1 − β) of 0.80. The analysis yielded a required total sample size of 30 animals, corresponding to six rats per group, with an actual power of 0.809. This sample size was also consistent with previous studies employing the letrozole-induced PCOS model [57,58].

### 3.4. Experimental Design

**Figure 13:**
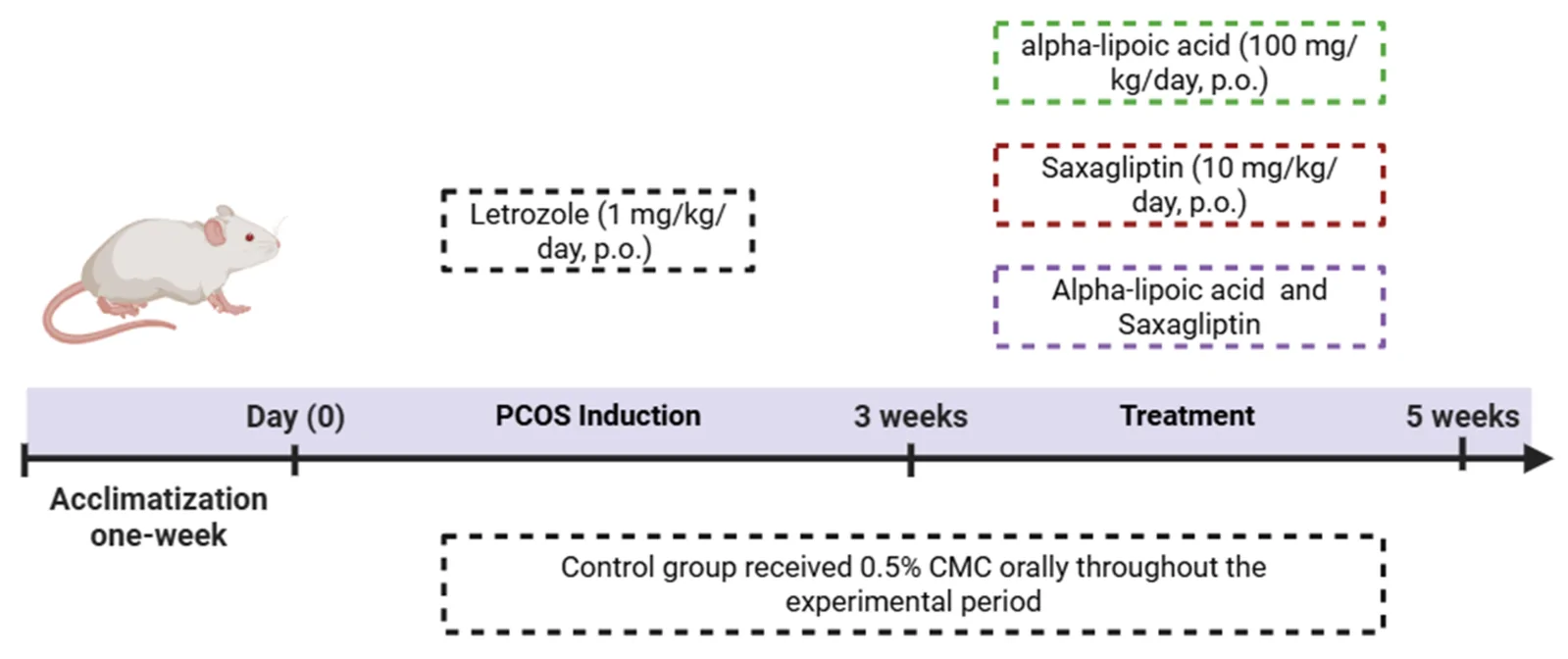
Schematic overview of the in vivo experimental research protocol.

Following a one-week acclimatization period, the experimental protocol extended over five weeks, as illustrated in the experimental timeline (Figure 13). Rats were assigned to either the control group or the letrozole-induced PCOS groups. PCOS was induced by daily oral administration of letrozole (1 mg/kg/day) suspended in 0.5% CMC for 21 consecutive days according to previously established protocols [59,60,61], whereas control rats received vehicle only. Animals were organized into five experimental groups (n = 6) and received their assigned oral treatments for an additional 14 days. Group 1 (Control): received 1 mL 0.5% CMC orally throughout the experimental period. Group 2 (PCOS): letrozole-induced PCOS rats that received 0.5% CMC orally by gavage for 14 days following the 21-day induction period, without active therapeutic treatment [59,60,61]. Group 3 (ALA): Letrozole-induced PCOS rats received ALA (100 mg/kg/day, p.o.), suspended in 1 mL of 0.5% CMC, for 14 days after the 21-day induction period [62,63]. Group 4 (Saxa): Letrozole-induced PCOS rats received Saxa (10 mg/kg/day, p.o.), suspended in 1 mL of 0.5% CMC, for 14 days after the 21-day induction period [64,65]. Group 5 (ALA/Saxa): Letrozole-induced PCOS rats received ALA (100 mg/kg/day, p.o.) and Saxa (10 mg/kg/day, p.o.) for 14 days after the 21-day induction period. The PCOS phenotype was subsequently evaluated based on body weight, ovarian index, ovarian histopathological changes, and reproductive hormonal alterations compared with the control group.

### 3.5. Sample Collection and Tissue Processing

At the end of the treatment period, rats were fasted overnight and anesthetized with pentobarbital sodium (40 mg/kg, i.p.). Blood samples were collected from the retro-orbital plexus, allowed to clot at room temperature, and centrifuged at 3000 rpm for 15 min to obtain serum, which was stored at −20 °C until analysis. After blood collection, animals were euthanized under deep anesthesia by exsanguination. Both ovaries were carefully excised, cleared of surrounding adipose tissue, blotted dry, and weighed. The ovarian index was calculated using the following formula [66]:

$$\mathrm{Ovarian index}=\frac{\mathrm{Ovarian weight}}{\mathrm{Body weight}}\times 100$$

The left ovary was fixed in 10% neutral buffered formalin for histopathological and immunohistochemical examination. The right ovary was divided into two portions; one portion was snap-frozen in liquid nitrogen and stored at −80 °C for Western blot analysis, while the remaining tissue was homogenized (10% *w*/*v*) in ice-cold phosphate-buffered saline (PBS, pH 7.4) using a Polytron homogenizer (KINEMATICA AG, Malters, Switzerland) maintained at 4 °C. Homogenates were centrifuged at 10,000 rpm for 15 min at 4 °C, and the resulting supernatants were collected and stored at −80 °C for biochemical assays, avoiding repeated freeze–thaw cycles.

### 3.6. Endocrine and Biochemical Analyses

Serum concentrations of testosterone, luteinizing hormone (LH), and follicle-stimulating hormone (FSH) were quantified using competitive inhibition rat ELISA kits obtained from CUSABIO (Wuhan, China; Cat. No. CSB-E05100r for T, CSB-E12654r for LH, and CSB-E06869r for FSH) according to the manufacturer’s instructions. Ovarian tissue homogenates were prepared under cold conditions, and levels of FGF21 (MBS2024083), NOX4 (MBS2503069), ROS (MBS039665), HO-1 (MBS764989), and GPX4 (MBS069787) were measured using rat-specific sandwich ELISA kits supplied by MyBioSource (San Diego, CA, USA) following the manufacturer’s protocols. Briefly, standards and diluted samples were added to pre-coated microplate wells and incubated for 1 h at 37 °C. After washing, the respective detection reagent was added: a direct HRP-conjugate for the competitive assays, incubated for 1 h at 37 °C; or, for the sandwich assays, a biotin-labeled detection antibody incubated for 1 h at 37 °C, followed by a further wash and incubation with avidin-HRP conjugate for 1 h at 37 °C. Plates were then washed and incubated with tetramethylbenzidine (TMB) substrate solution for 15–20 min at 37 °C in the dark, the reaction was stopped with stop solution, and absorbance was read at 450 nm using a microplate reader. Concentrations were calculated from the corresponding standard curves. In addition, SLC7A11 protein expression was assessed by Western blot analysis as an independent protein-based validation of the ferroptosis-related changes. MDA was quantified using a colorimetric assay kit from Bio-Diagnostics (Giza, Egypt; Cat. No. MD2529). Ferrous ion (Fe^2+^) content was determined using a colorimetric assay kit (BioVision, Milpitas, CA, USA; Cat. No. K390-100) according to the manufacturer’s instructions, in which Fe^2+^ present in the sample reacted directly with Ferene S to produce a stable-colored complex. Ovarian tissue was homogenized in Iron Assay Buffer (10% *w*/*v*) and centrifuged at 16,000× *g* for 10 min at 4 °C, and the supernatant was collected. Samples and Fe^2+^ standards (100 µL/well) were incubated for 30 min at 37 °C, followed by 100 µL Iron Probe (Ferene S reagent) per well and a further 60 min incubation at 37 °C protected from light. Absorbance was read at 593 nm, and Fe^2+^ concentrations were calculated from the standard curve. The protein concentration was determined by Bradford assay (Bio-Rad Laboratories, Inc., Hercules, CA, USA) using a microplate reader (Bio-Rad) at 595 nm. Then, the ovarian ELISA results and MDA and Fe^2+^ concentrations were normalized to total ovarian protein. All assays were performed in duplicate, and mean values were used for statistical analyses to ensure accuracy, reliability, and reproducibility.

### 3.7. Ovarian Histopathology

Before embedding, the fixed ovary was cut along its axial direction into 2 halves, fixed in 10% neutral buffered formalin, and routinely processed. Ovaries were passed through a graded alcohol series, cleared in xylene, and embedded in paraffin blocks. Sections of 5 µm thickness were cut using a rotary microtome and stained with hematoxylin and eosin (H&E): deparaffinized sections were rehydrated through a descending ethanol series, stained with Mayer’s hematoxylin for 5 min, differentiated in acid alcohol, blued in running tap water, counterstained with eosin Y for 2 min, dehydrated through graded ethanol, cleared in xylene, and mounted with DPX. The assessment of follicular quality was based on cellular density and the integrity of the basement membrane and the oocyte; only morphologically normal follicles were quantified according to these criteria. Follicles were classified in the histological preparations, based on stage, into primordial, primary, secondary, or antral (Graafian) follicles. Briefly, primordial follicles were defined as an oocyte surrounded by a single epithelial cell layer; primary follicles were characterized by a single cell layer of cubic or high-prismatic cells; secondary follicles consisted of multiple layers of granulosa cells; and Graafian follicles were defined by the presence of an antrum folliculi, stratum granulosum, and cumulus oophorus. Quantitative analysis included enumeration of primordial, primary, secondary, and mature follicles, as well as atretic and cystic follicles and corpora lutea. Total follicle counts excluded corpora lutea, as previously described [67]. Each follicle type was counted only once per entire ovarian section per animal to avoid double-counting of large follicles or corpora lutea, as described by Ozdamar et al. [68]. Stromal edema was semi-quantitatively scored on a 0–3 scale, with 0 indicating no edema and 1, 2, and 3 representing edema affecting <33%, 33–66%, and >66% of the ovarian section, respectively [68,69]. All measurements were performed by a histopathologist blinded to experimental groups, and stained sections were examined and imaged using a Leica DFC imaging system (Leica Microsystems GmbH, Wetzlar, Germany).

### 3.8. Immunohistochemical Labeling of NRF2 in Ovarian Tissue

The expression of nuclear factor erythroid 2-related factor 2 (NRF2) in ovarian tissue was assessed immunohistochemically. Paraffin-embedded ovarian tissues were sectioned at 5 µm thickness and mounted on glass slides, followed by deparaffinization in xylene and rehydration through graded ethanol. Heat-induced antigen retrieval was performed in 10 mM citrate buffer (pH 6.0), endogenous peroxidase activity was quenched with 3% hydrogen peroxide, and nonspecific binding was blocked using an appropriate protein blocking solution. Sections were then incubated with the anti-NRF2 primary antibody (Cat. No. GTX55732; GeneTex, Irvine, CA, USA) at a dilution of 1:200 overnight at 4 °C, followed by incubation with an HRP-conjugated goat anti-rabbit IgG secondary antibody (Cat. No. GTX213110-01; GeneTex, Irvine, CA, USA) at a dilution of 1:500 for 1 h at room temperature. Immunoreactivity was visualized using a DAB substrate kit (3,3′-diaminobenzidine; Abcam, Cambridge, UK, Cat. No. ab64238), with color development monitored under a light microscope for 3 min. Sections were counterstained with Mayer’s hematoxylin for 2 min, dehydrated through graded ethanol, cleared in xylene, and mounted with DPX. Immunostained sections were examined under a light microscope, and representative images were captured at ×400 under identical illumination and exposure settings using a Leica DFC camera system. NRF2 immunoreactivity appeared as brown DAB staining. Quantitative image analysis was performed in ImageJ (version 1.53; National Institutes of Health, Bethesda, MD, USA) using the Color Deconvolution plugin (HDAB vector) to separate the DAB signal from the hematoxylin counterstain [70]. DAB-positive staining was isolated with a single fixed threshold applied identically to all images [71]. For each of five ovarian sections per group, randomly selected, non-overlapping high-power fields were analyzed, and the percentage of positively stained area relative to the total tissue area was calculated. Analysis was performed by an observer blinded to group allocation.

### 3.9. Immunoblot Analysis of SLC7A11 Expression in Ovarian Tissue

Ovarian tissues were homogenized in ice-cold RIPA lysis buffer supplemented with protease and phosphatase inhibitors and centrifuged at 12,000× *g* for 15 min at 4 °C. Total protein concentrations were determined using a bicinchoninic acid (BCA) protein assay kit (Thermo Fisher Scientific, Rockford, IL, USA). Equal amounts of protein (30–40 µg) were denatured in Laemmli sample buffer, separated by 10–12% SDS-PAGE, and electrotransferred onto PVDF membranes. Membranes were blocked with 5% non-fat milk in TBST for 1 h at room temperature and incubated overnight at 4 °C with primary antibodies against SLC7A11 (Invitrogen/Thermo Fisher Scientific; Cat. No. PA1-16893; 1:1000) and β-actin (Invitrogen/Thermo Fisher Scientific; Cat. No. MA1-140; clone 15G5A11/E2; 1:5000). Following TBST washes, membranes were incubated for 1 h at room temperature with an HRP-conjugated goat anti-rabbit IgG secondary antibody (Cat. No. GTX213110-01; GeneTex, Irvine, CA, USA) at a dilution of 1:5000 to detect SLC7A11, or an HRP-conjugated goat anti-mouse IgG secondary antibody (Cat. No. GTX213111-01; GeneTex, Irvine, CA, USA) at a dilution of 1:5000 to detect β-actin. Immunoreactive bands were detected using enhanced chemiluminescence and quantified by densitometric analysis with ImageJ software (version 1.53; National Institutes of Health, Bethesda, MD, USA), with SLC7A11 expression normalized to β-actin.

### 3.10. Statistical Analysis

Data distribution was assessed for normality prior to statistical testing. Normally distributed data are expressed as mean ± standard error of the mean (SEM). Comparisons among multiple groups were performed using one-way ANOVA followed by Tukey’s post hoc test. Histopathological data, which were treated as non-parametric, are presented as median and interquartile range (IQR), with whiskers indicating the minimum and maximum, and were analyzed using the Kruskal–Wallis test followed by Dunn’s multiple-comparisons test. Statistical analysis and graphical representations were conducted using GraphPad Prism (version 10.4.1; GraphPad Software, San Diego, CA, USA). A two-tailed *p*-value ˂ 0.05 was considered statistically significant. Hierarchical clustering and heatmap analysis were performed using the online ChiPlot platform (https://www.chiplot.online/ accessed on 20 May 2026). Prior to heatmap generation, all variables were standardized by row-wise Z-score normalization to eliminate differences in measurement scales among the measured parameters.

## 4. Discussion

PCOS is a heterogeneous endocrine-metabolic disorder characterized by reproductive, hormonal, and metabolic abnormalities, although the molecular mechanisms linking metabolic dysfunction to ovarian injury remain incompletely understood [72]. Insulin resistance is considered a central contributor to PCOS pathogenesis and is closely associated with many of its metabolic complications [73]. Increasing evidence also implicates iron overload and ferroptosis in metabolic dysfunction, impaired glucose tolerance, and diabetes-related complications [74]. In PCOS, elevated ferritin levels and increased reactive oxygen species (ROS) production further support the involvement of iron-dependent oxidative injury in disease progression [75]. However, therapeutic strategies targeting ferroptosis-related ovarian damage in PCOS remain insufficiently explored. Therefore, the present study investigated whether ALA, Saxa, and particularly their combination could attenuate ferroptosis-related ovarian injury in letrozole-induced PCOS.

Ferroptosis is associated with mitochondrial dysfunction, oxidative stress, and lipid peroxidation [76]. Consistent with this evidence, the PCOS group exhibited significantly elevated ovarian Fe^2+^, ROS, and MDA levels, indicating iron accumulation, oxidative injury, and enhanced lipid peroxidation. These findings are consistent with previous evidence showing mitochondrial dysfunction and ferroptosis-related ovarian injury in experimental PCOS model [17]. Together, these alterations suggest that letrozole-induced PCOS is accompanied by a redox imbalance that favors ferroptosis-related ovarian damage. However, it should be acknowledged that MDA reflects overall lipid peroxidation, whereas ROS represents a broader index of oxidative stress rather than a ferroptosis-specific measure of lipid ROS. Therefore, although the observed changes in Fe^2+^, ROS, and MDA are consistent with ferroptosis-related alterations, they do not independently establish ferroptotic cell death. Future studies incorporating direct assessment of lipid ROS, additional ferroptosis-associated markers such as ACSL4 and TFRC, and ferroptosis-specific rescue approaches using agents such as ferrostatin-1 are warranted to confirm the involvement of ferroptosis in this model.

NOX4 appears to be an important upstream contributor to this oxidative imbalance. As a member of the NADPH oxidase family, NOX4 is localized to several cellular organelles, including mitochondria, and contributes to ROS generation, transcriptional regulation, proliferation, migration, and cell death [77]. Previous studies reported increased ovarian NOX4 expression in PCOS rat models, whereas NOX4 deficiency improved PCOS-related cellular injury [26,78]. In agreement, the present study showed significantly increased ovarian NOX4 levels in the PCOS group compared with control group. Considering that NOX4 is a major source of intracellular ROS, its upregulation has been associated with lipid peroxidation and ferroptotic cell death [27,79], thereby suggesting an association between NOX4 upregulation and ferroptosis susceptibility in PCOS.

In parallel, the PCOS group showed a marked reduction in ovarian FGF21 levels. More recently, FGF21 has been identified as a negative regulator of ferroptosis [24], with reported anti-ferroptotic effects involving attenuation of lipid-derived ROS accumulation and restoration of mitochondrial function [80]. Thus, reduced ovarian FGF21 in the PCOS group may reflect a loss of metabolic and redox-adaptive signaling that normally limits oxidative and ferroptosis-related injury. This observation supports the relevance of FGF21 as a potential counter-regulatory factor in PCOS-associated ovarian dysfunction. However, ovarian FGF21 was measured in ovarian homogenates by ELISA, so locally produced and circulating FGF21 cannot be distinguished, and systemic metabolic parameters were not assessed. Future studies combining ovarian FGF21 expression and localization with circulating FGF21 and systemic metabolic profiling would clarify the broader metabolic relevance of these findings.

The decline in FGF21 was accompanied by suppression of NRF2 signaling. FGF21 -associated regulation of ferroptosis has been reported to involve activation of ERK/NRF2 signaling, a major antioxidant pathway that enhances redox defense and limits lipid peroxidation [25,81]. In the present study, NRF2 immunoreactivity was markedly reduced in PCOS ovaries, supporting an association between reduced FGF21 levels, reduced antioxidant defense, and ferroptosis-related injury. Downstream NRF2-regulated effectors were similarly reduced, as HO-1, GPX4, and SLC7A11 levels were significantly decreased in the PCOS group. HO-1 contributes to antioxidant defense [82], GPX4 detoxifies lipid peroxides and limits ferroptosis [83], and SLC7A11 maintains cystine uptake and glutathione homeostasis [84]. These findings are consistent with prior reports implicating NRF2-regulated antioxidant responses in protection against oxidative stress and ferroptotic cell death [22,85,86]. Although HO-1 and GPX4 were quantified using validated sandwich ELISA assays and SLC7A11 was additionally assessed by Western blot, the present study did not perform Western blot validation for all ferroptosis-related protein markers. Therefore, further studies using complementary protein-based approaches are warranted to confirm these findings and provide additional mechanistic validation.

These biochemical and molecular alterations were reflected in ovarian histopathological changes and endocrine disturbance. Histopathological examination showed that PCOS ovaries exhibited reduced numbers of primordial, primary, secondary, and mature follicles, as well as corpora lutea, along with increased atretic follicles, cystic follicles, and stromal edema. In addition, the PCOS group exhibited reproductive hormonal imbalance, including elevated testosterone, LH, and LH/FSH ratio, together with reduced FSH. Because mitochondria are central to ovarian steroid hormone biosynthesis [87], these findings suggest that ferroptosis-related mitochondrial and redox dysfunction may contribute to impaired follicular development and endocrine disruption in letrozole-induced PCOS. Notably, PCOS induction was not confirmed before treatment (e.g., by estrous cyclicity); however, the endpoint hormonal and histopathological alterations in the untreated PCOS group support successful model establishment.

Given the multifactorial nature of PCOS, combination strategies that target complementary metabolic and redox pathways may offer broader therapeutic benefit. ALA is a natural antioxidant and mitochondrial modulator [88], whereas Saxa improves insulin sensitivity and metabolic regulation [89]. In the present study, ALA, Saxa, and their combination improved reproductive hormonal imbalance, body weight, ovarian index, and ovarian histopathology compared with untreated PCOS rats. Importantly, the combined ALA/Saxa treatment produced greater improvement across ovarian histopathological, endocrine, oxidative, and ferroptosis-related parameters. However, this study was not designed to assess pharmacological synergy; the greater effects of the combination should be interpreted as enhanced combined effects rather than synergism, which would require dose–response and drug-interaction studies.

The beneficial effects of ALA/Saxa were closely associated with restoration of redox balance. ALA, Saxa, and their combination reduced ovarian ROS and MDA levels, consistent with previous studies showing that ALA [90] and Saxa [91] alleviate oxidative stress and support mitochondrial repair in experimental rodent models. In parallel, all treated groups showed reduced NOX4 levels, with the combination producing a greater reduction than either monotherapy. This finding aligns with previous reports showing that NOX4 inhibition reduces ROS production and oxidative injury in experimental models of diabetes and intracerebral hemorrhage [92,93]. These results support the association between reduced NOX4 levels and reduced oxidative stress in ALA/Saxa-treated PCOS rats. However, fluorescence-based ROS assessment would complement the ELISA data, and targeted pharmacological or genetic manipulation of FGF21 and NOX4 is needed to establish their causal involvement.

ALA/Saxa treatment was also associated with enhanced FGF21 and NRF2/HO-1 signaling. Previous studies showed that FGF21 administration in rodents alleviates obesity-related metabolic complications by reducing oxidative stress and lipid peroxidation [94]. In agreement with this evidence, ALA, Saxa, and ALA/Saxa-treated groups exhibited increased ovarian FGF21 levels compared with untreated PCOS rats. NRF2 immunoreactivity was also markedly increased, consistent with previous evidence suggesting a positive association between FGF21 and NRF2 signaling in atherosclerotic rat models [95]. Downstream antioxidant and ferroptosis-related markers were similarly improved, including HO-1, GPX4, and SLC7A11. These findings suggest that ALA/Saxa treatment was associated with coordinated improvement in antioxidant defense and ferroptosis-related markers. However, NRF2 was assessed by immunohistochemistry and therefore reflects immunoreactivity rather than nuclear translocation or transcriptional activation; nuclear/cytoplasmic fractionation would better evidence pathway activation.

To explore potential molecular interactions relevant to the observed protective effects of ALA and Saxa, molecular docking studies were performed against several upstream regulators implicated in PCOS pathogenesis, including IKKβ, SIRT1, PPAR-α, AMPK, and KEAP1. Although FGF21 and NOX4 were experimentally evaluated in the present study, their available structural information is not ideally suited for conventional small-molecule docking. Therefore, proteins implicated in pathways related to FGF21/NOX4 and antioxidant regulation were selected.

Accumulating evidence indicates that chronic low-grade inflammation contributes to ovarian dysfunction and metabolic disturbances in PCOS [96,97]. Activated IKKβ promotes NF-κB signaling, resulting in increased production of pro-inflammatory mediators and ROS [98], which subsequently stimulate NOX4 expression and oxidative stress [99]. Previous studies have shown that inhibition of NF-κB signaling alleviates ovarian inflammation and improves endocrine abnormalities in experimental PCOS models [100]. In the present study, the favorable binding of both ALA and Saxa within the IKKβ active site suggests potential interactions that may be relevant to the observed reduction in NOX4 and oxidative stress markers.

The AMPK/SIRT1 signaling axis is a key regulator of metabolic homeostasis, orchestrating glucose and lipid metabolism while suppressing inflammation and improving insulin sensitivity [97,101]. Activation of this pathway has been reported to enhance the transcriptional activity of PPAR-α, a nuclear receptor that plays a pivotal role in fatty acid oxidation, glucose utilization, and the maintenance of metabolic balance [52,102,103]. Notably, PPAR-α activation has been associated with improved insulin resistance and ovarian function in PCOS [104] and is recognized as a major upstream regulator of FGF21 expression [52]. In the present study, treatment with ALA, Saxa, or their combination restored ovarian FGF21 levels, while molecular docking analyses predicted potential binding interactions of both compounds with AMPK, SIRT1, and PPAR-α. These docking findings are hypothesis-generating and are consistent with previous studies in rodent PCOS models in which AMPK phosphorylation and downstream signaling components were experimentally assessed to investigate the involvement of the AMPK/SIRT1/PPAR-α axis [105,106]. Because ALA and Saxa were docked independently, whether their predicted interactions at overlapping sites are competitive or cooperative in vivo remains unresolved, and direct assessment of AMPK/SIRT1/PPAR-α signaling is required.

Oxidative stress and ferroptosis have recently been recognized as critical contributors to follicular dysfunction in PCOS [100,107]. The KEAP1/NRF2 pathway serves as a major cellular defense system against oxidative injury by inducing antioxidant and ferroptosis-suppressing proteins, including HO-1, GPX4, and SLC7A11 [108]. Under physiological conditions, KEAP1 sequesters NRF2 in the cytoplasm and promotes its degradation. Disruption of the KEAP1– NRF2 interaction facilitates NRF2 nuclear translocation and activation of antioxidant gene expression [109]. In agreement with previous reports demonstrating the protective role of NRF2 activation in PCOS, our results showed marked increases in NRF2, HO-1, GPX4, and SLC7A11 following treatment. The predicted docking interactions with KEAP1 suggest potential interactions that may be relevant to the observed changes in NRF2 and ferroptosis-related markers. As the docking analyses were predictive only, direct assessment of KEAP1–NRF2 interaction and signaling is required to confirm these interactions.

Collectively, ALA and Saxa, particularly in combination, improved ovarian morphology, reproductive hormonal imbalance, oxidative stress, and ferroptosis-related disturbances in letrozole-induced PCOS rats. These effects were associated with increased ovarian FGF21 expression, reduced NOX4 levels, increased NRF2/HO-1-related antioxidant responses, and preservation of GPX4/SLC7A11. Thus, ALA/Saxa may improve ovarian dysfunction by coordinating redox and ferroptosis-related pathways. However, further mechanistic studies using pathway-specific inhibition, knockdown, or rescue approaches are required to establish causality, and translational studies are needed to assess clinical relevance.

## 5. Conclusions

The current study demonstrates that Saxa and ALA, especially in combination, ameliorated the ovarian, hormonal, oxidative and ferroptosis-related alterations induced by letrozole-induced PCOS in rats. The combination treatment resulted in greater improvements in ovarian morphology and reproductive hormonal balance, concomitant with reduced oxidative stress and ferroptosis-related alterations. These beneficial effects were associated with increased ovarian FGF21 and reduced NOX4 levels, increased NRF2/HO-1-related antioxidant responses, and preservation of GPX4 and SLC7A11. To summarize, these findings support the potential of ALA/Saxa as a multi-target strategy for mitigating ovarian dysfunction associated with PCOS in association with modulation of metabolic, redox and ferroptosis-related pathways.

## Figures and Tables

**Table 1:** Molecular interactions of Target Proteins and Their Co-crystallized Ligands.

Molecular Target	Cocrystallized Ligand	Hydrogen Bond Analysis		Amino Acids Involved in Lipophilic Analysis
		Amino Acid Involved in H Bond	Distance (Å)	
IKKβ Chain A	XNM	Lys106	2.68289	Val29, Ala42, Met96, Cys99, Val152, and Ile165
		Asp103	2.94909	
		Leu21	2.29479	
SIRT1 chain A	4TQ	Asn226	1.67356	Ile223, Ile227, and Pro212
PPARα Chain A	T02	Ser280	1.89182	Ile272, Phe273, Cys276, Leu321, Leu344, Leu347, Ile354, Met355, Val444, and His440
		Tyr314	1.61372	
		His440	1.58409	
		Tyr464	1.69914	
AMPK chain c	C1V	Lys31	2.18612	Ile46, Val11
		Lys31	2.26941	
		Gly19	1.68101	
AMPK chain e	C2Z	Ser314	1.69386	His151
		His151	1.85064	
		Ser226	2.29351	
		Arg299	2.65671	
		His298	2.79032	
AMPK chain F	AMP	Asp245	2.05884	Val297, Ile240, Leu277
		Asp245	1.89406	
		His298	2.19441	
		Ser242	2.17557	
		Arg269	2.98464	
		Arg269	2.71296	
		Leu277	2.25135	
		Leu277	2.44659	
		Arg70	2.30963	
		Arg299	2.72413	
		Arg299	2.02352	
		Arg299	2.58342	
		Arg299	2.21486	
KEAP1 Chain A	SXJ	-	-	Lys131, Cys151, Val155, His129, and His154

**Table 2:** Binding affinities and molecular interactions of ALA and Saxa with target proteins.

Molecular Target	Ligand	Hydrogen Bond Analysis		Amino Acids Involved in Lipophilic Analysis	Binding Affinity (kcal/mol)
		Amino Acid Involved in H Bond	Distance (Å)		
IKKβ Chain A	ALA	CYS99	2.15052	Ala42, Leu21, Ile165, Val29, and Tyr98	−5.1
		GLY102	2.2302		
	Saxa	GLU97	2.60558	Leu21, Val29, Ala42, Tyr98, Cys99, Val152, and Ile165	−7.4
		CYS99	3.09841		
		GLU149	3.05497		
		GLU149	2.97617		
SIRT1 chain A	ALA	ASN226	2.06285	Ile227	−3.3
	Saxa	ASN226	2.52907	Ile223 and Ile227	−4.7
PPARα Chain A	ALA	SER280	2.25354	Cys276, Ile354, and Phe273	−5.5
		TYR314	2.28484		
		HIS440	2.16618		
		TYR464	1.98069		
	Saxa	SER280	2.09593	Cys276, Leu321, Ile354, Phe273, and His440.	−4.9
		TYR314	2.41037		
		TYR464	2.27076		
		MET355	3.04024		
AMPK chain c	ALA	GLY28	2.71424	Ile46, Lys29	−3.7
		ASN48	1.98555		
	Saxa	ASN48	3.0714	Ile46, Lys29, Phe90	−5.2
		ASN48	2.73788		
		ASP88	2.403		
AMPK chain e	ALA	ILE88	2.28178	Lys243, Ile88, His151	−5.5
		THR89	2.1933		
		THR89	1.87224		
		ARG152	2.78836		
	Saxa	HIS151	2.16382	Ile312, Val225, Ile204	−7.5
		SER226	2.40848		
		ALA227	2.18881		
		ARG299	1.94829		
		SER314	2.85137		
AMPK chain f	ALA	ARG70	2.21744	Val297 and Ile240	−5
		LYS170	2.94908		
		LYS170	2.70564		
		SER242	2.17131		
		HIS298	2.32431		
	Saxa	LYS170	2.3374	Phe244, Ile240, Val297, and Leu277	−7.3
		HIS298	2.85174		
		ARG299	2.25583		
		ARG299	2.39751		
KEAP1 Chain A	ALA	-	-	Lys131, Val132, Cys151, Tyr85, and His154	−4.2
	Saxa	Gln86	2.42596	Ala88, Cys151, Tyr85, and His154.	−5.2

**Table 3:** Grid box dimension used for docking.

Protein	PDB Code	Co-Crystallized Ligand	Dimension		
			X	Y	Z
AMPK chain c	4ZHX	C1V	163.130125	−37.411475	69.238500
AMPK chain e		C2Z	76.970158	14.525211	30.156211
AMPK chain f		AMP	120.463029	−90.128543	34.372200
PPARα Chain A	6KXX	T02	14.351667	−12.232854	−30.358417
SIRT1 chain A	4ZZJ	4TQ	−0.423893	44.524196	−0.038714
IKKβ Chain A	3RZF	XNM	90.947069	−22.526534	54.620690
KEAP1 Chain A	4CXT	SXJ	0.607987	5.242390	−8.224584

## Data Availability

All data and materials in our study are available upon reasonable request.
